# 2b-RAD genotyping for population genomic studies of Chagas disease vectors: *Rhodnius ecuadoriensis* in Ecuador

**DOI:** 10.1371/journal.pntd.0005710

**Published:** 2017-07-19

**Authors:** Luis E. Hernandez-Castro, Marta Paterno, Anita G. Villacís, Björn Andersson, Jaime A. Costales, Michele De Noia, Sofía Ocaña-Mayorga, Cesar A. Yumiseva, Mario J. Grijalva, Martin S. Llewellyn

**Affiliations:** 1 Institute of Biodiversity, Animal Health and Comparative Medicine, University of Glasgow, Glasgow, United Kingdom; 2 Department of Biology, University of Padua, Padua, Italy; 3 Consorzio Nazionale Interuniversitario per le Scienze del Mare (CoNISMa), Rome, Italy; 4 Center for Research on Health in Latin America, School of Biological Sciences, Pontifical Catholic University of Ecuador, Quito, Ecuador; 5 Department of Cell and Molecular Biology, Karolinska Institutet, Stockholm, Sweden; 6 Department of Animal Behaviour, Bielefeld University, Bielefeld, Germany; 7 Infectious and Tropical Disease Institute, Department of Biomedical Sciences, Heritage College of Osteopathic Medicine, Ohio University, Ohio, United States of America; New York University, UNITED STATES

## Abstract

**Background:**

*Rhodnius ecuadoriensis* is the main triatomine vector of Chagas disease, American trypanosomiasis, in Southern Ecuador and Northern Peru. Genomic approaches and next generation sequencing technologies have become powerful tools for investigating population diversity and structure which is a key consideration for vector control. Here we assess the effectiveness of three different 2b restriction site-associated DNA (2b-RAD) genotyping strategies in *R*. *ecuadoriensis* to provide sufficient genomic resolution to tease apart microevolutionary processes and undertake some pilot population genomic analyses.

**Methodology/Principal findings:**

The 2b-RAD protocol was carried out in-house at a non-specialized laboratory using 20 *R*. *ecuadoriensis* adults collected from the central coast and southern Andean region of Ecuador, from June 2006 to July 2013. 2b-RAD sequencing data was performed on an Illumina MiSeq instrument and analyzed with the STACKS *de novo* pipeline for loci assembly and Single Nucleotide Polymorphism (SNP) discovery. Preliminary population genomic analyses (global AMOVA and Bayesian clustering) were implemented. Our results showed that the 2b-RAD genotyping protocol is effective for *R*. *ecuadoriensis* and likely for other triatomine species. However, only BcgI and CspCI restriction enzymes provided a number of markers suitable for population genomic analysis at the read depth we generated. Our preliminary genomic analyses detected a signal of genetic structuring across the study area.

**Conclusions/Significance:**

Our findings suggest that 2b-RAD genotyping is both a cost effective and methodologically simple approach for generating high resolution genomic data for Chagas disease vectors with the power to distinguish between different vector populations at epidemiologically relevant scales. As such, 2b-RAD represents a powerful tool in the hands of medical entomologists with limited access to specialized molecular biological equipment.

## Author summary

Understanding Chagas disease vector (triatomine) population dispersal is key for the design of control measures tailored for the epidemiological situation of a particular region. In Ecuador, *Rhodnius ecuadoriensis* is a cause of concern for Chagas disease transmission, since it is widely distributed from the central coast to southern Ecuador. Here, a genome-wide sequencing (2b-RAD) approach was performed in 20 specimens from four communities from Manabí (central coast) and Loja (southern) provinces of Ecuador, and the effectiveness of three type IIB restriction enzymes was assessed. The findings of this study show that this genotyping methodology is cost effective in *R*. *ecuadoriensis* and likely in other triatomine species. In addition, preliminary population genomic analysis results detected a signal of population structure among geographically distinct communities and genetic variability within communities. As such, 2b-RAD shows significant promise as a relatively low-tech solution for determination of vector population genomics, dynamics, and spread.

## Introduction

Vector control has been the mainstay of Chagas disease control strategies in Latin America. Several Latin American countries implemented nation-wide insecticide-spraying programs to eradicate Chagas disease vector populations in human dwellings over the last 30 years. These campaigns resulted in a dramatic reduction in vectorial transmission [1–3]. Despite this success, domicile recolonization is a constant threat due to the ability of several triatomines species to disperse from sylvatic to domestic/peridomestic environments and establish local domestic populations [4–8].

Triatomines, members of the arthropod family Reduviidae, subfamily Triatominae, commonly known as kissing bugs, are distributed from the southern United States to central Argentina [9]. Over 130 species are identified, but only a few dozen are known to transmit Chagas disease [10]. In Ecuador, *Triatoma dimidiata* and *Rhodnius ecuadoriensis* are main vectors of Chagas disease, with the latter widely distributed from coastal and southern Ecuador to northern Peru [11,12].

Multiple molecular genetic studies exist which attempt to explain genetic structure and gene flow in triatomine populations [8,13–20]. An example of those tailored to address defined epidemiological hypotheses include that of Fitzpatrick *et al*. [13]. Fitzpatrick *et al*. confirmed that gene flow (and therefore vector dispersal) occurs between sylvatic, domicile and peridomicile ecotopes in Venezuelan *Rhodnius prolixus* based on pairwise F_ST_ values derived from both cytochrome b (*cytb*) and nine microsatellite loci. *R*. *prolixus* is the major vector species in Venezuela and Colombia, as well as Andean and Central American countries. Fitzpatrick *et al*.’s data suggested that colonization of domestic locales by wild triatomines is indeed possible in the region, and these findings had major implications for control. Other species have also been the subject of study. Population genetic data from *Triatoma infestans* based on ten microsatellite loci showed fine-scale genetic structure in domestic populations several years after the spraying of insecticides [18]. In this case, genetic data were tested under two different models of dispersal: isolation by distance and hierarchical island with stratified migration. The latter best reflected vector genetic structure among the sample sites. Finally, Almeida and colleagues [20] compared *cytb* and 8 microsatellite loci in *Triatoma brasiliensis* to investigate its genetic structure and to assess gene flow among sylvatic and domestic/peridomestic populations. As with Fitzpatrick *et al*. [13], pairwise comparison of F_ST_ values obtained from microsatellite loci analysis also demonstrated connectivity between locales.

Given that vector control remains the mainstay of Chagas disease intervention strategies, greater understanding of vector genetics and dispersal is urgently required. Of particular importance are genotyping approaches that provide very high resolution at local, epidemiologically relevant scales, as well as the ability to share and combine datasets across different studies and research groups. Microsatellite loci offer little flexibility in terms of shareability as data standardization guidelines for amplicon size estimation and allele nomenclature between laboratories, although possible [21], are rarely established, time-consuming and expensive to resolve, an issue already seen in *Trypanosoma cruzi* typing [22]. Likely as a function of funding constraints, molecular genetic research on triatomine vectors, and on Chagas disease in general, has been relatively late to arrive on the ‘omics’ scene. The belated publication of *R*. *prolixus* genome in 2015, as compared to other vector species, represents a step in the right direction and has revealed much about the core adaptations that underpin the biological success of triatomines [23]. A number of expressed sequence tags have been developed for *T*. *infestans* [24,25]. However, in general, genome sequencing efforts in triatomines so far have yielded little benefit to scientists and public health professionals attempting to map vector dispersal.

In tandem with the emergence of high throughput next generation sequencing (NGS) approaches, several groups have pioneered the use of restriction enzymes (REases) on restriction site-associated DNA sequencing (RADseq) protocols to allow a small fraction of the genome to be sequenced across multiple samples [26–34]. Several variants of the RADseq technique currently exist [35–39]; however, protocol choice to address a specific research question must balance technical issues, budget and laboratory capacity [40].

The 2b-RAD genotyping strategy specifically uses Type IIB restriction enzymes (IIB-REases) for genomic DNA (gDNA) digestion [38]. Advantages of this protocol include simplicity and cost-efficiency, since it is carried out in 3 steps in the same 96-well plate, as compared to 4–6 steps required in other RADseq protocols [35–37, 39]. Furthermore, library preparation can be achieved with no more than a PCR machine and a standard agarose gel. Moreover, IIB-REases capacity to generate identically sized 2b-RAD tags (IIB-REase-dependent) across all samples [38,40] and cleave at both strands of DNA removes the need for a post-digestion fragment size selection step. These characteristics also prevent fragment size [41] and strand [42] sequencing bias, which can compromise genotyping calls, as seen in other RADseq protocols. One disadvantage compared to other RADseq methods is that 2b-RAD may be inappropriate where accurate mapping against a highly duplicated/polyploid reference genome is required due to short fragment size production (33–36 bp) [43]. Finally, bias from PCR duplicates, sequencing errors and allele dropout can be introduced in all RADseq protocols.

In our study, we were able to rapidly and cost-effectively generate several hundred Single Nucleotide Polymorphism (SNP) markers for *R*. *ecuadoriensis* allowing for resolution of regional population genetic structure. Furthermore, by comparing the performance among the three IIB-REases, we were able to recommend the appropriate IIB-REase and read depth to employ in order to yield a given number of SNP markers for *R*. *ecuadoriensis*, and presumably for other members of the *Rhodnius* genus.

## Methods

### Sample collection and gDNA extraction

**Fig 1:**
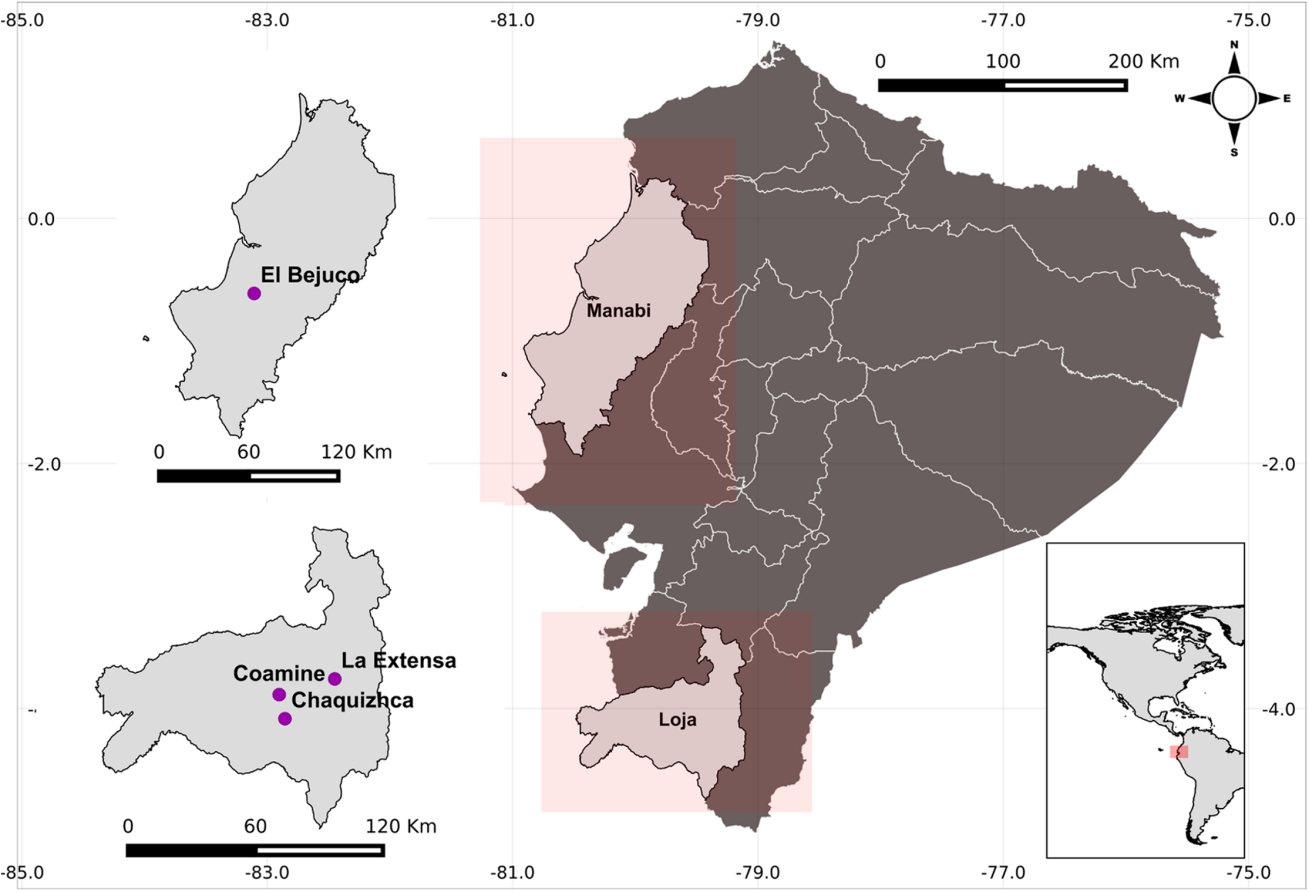
Map of the study area and the location of sampled communities in Ecuador. Purple circles indicate the location of Coamine (CE), La Extensa (EX) and Chaquizhca (CQ) in Loja Province, and El Bejuco (BJ) in Manabí.

A total of 20 samples of *R*. *ecuadoriensis* were selected from the communities of La Extensa, Chaquizhca, and Coamine in Loja Province (southern Andean region), and from the community of Bejuco in Manabí (central coast) in Ecuador (Fig 1). Triatomines were captured in previous field surveys [44–46] from June 2006 to July 2013 (see S1 Table for further sample information). For each sample, head, legs and thoraxes were dissected and preserved in 100% ethanol.

**Fig 2:**
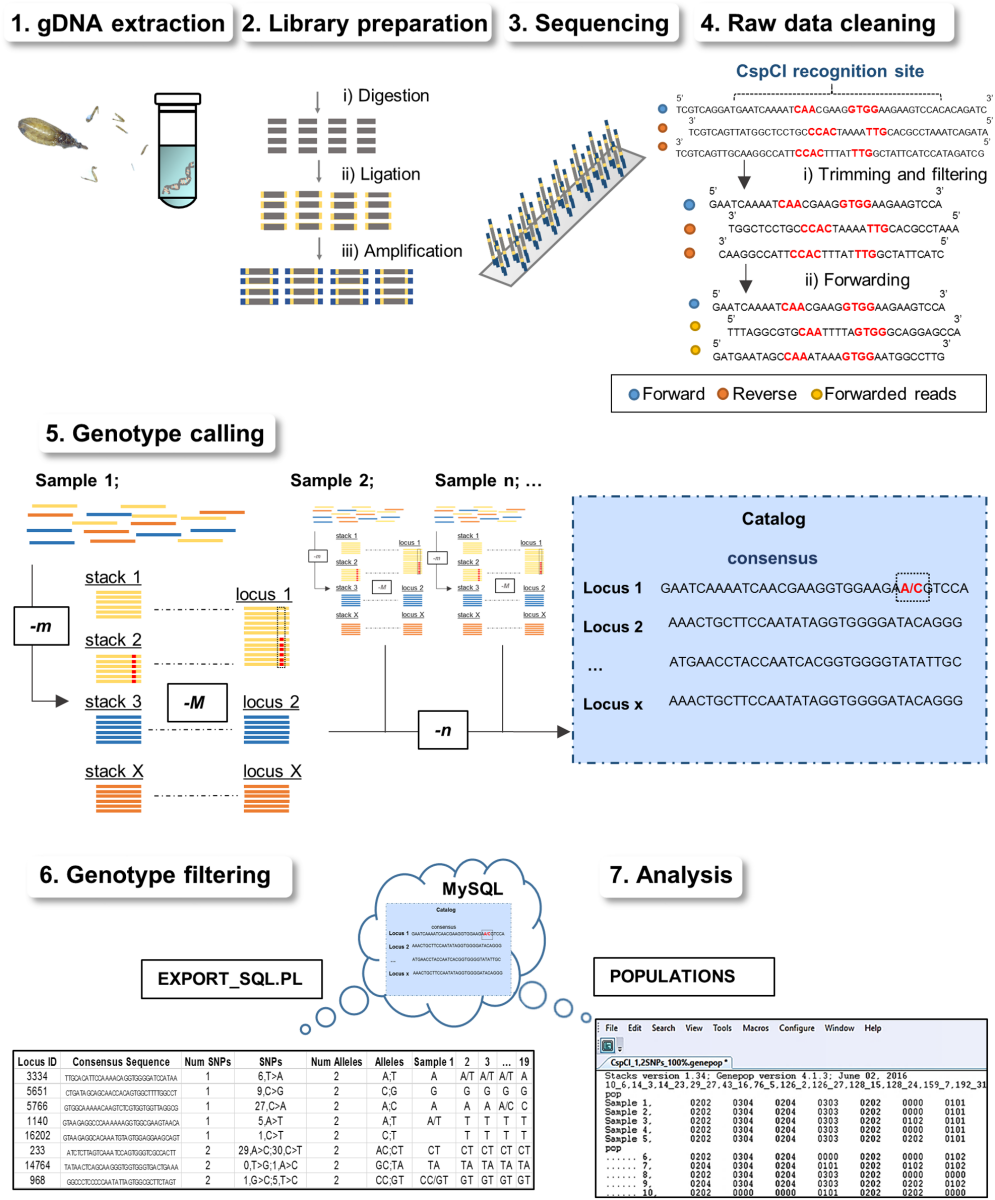
Step-by-step of 2b-RAD library and genomic data preparation for triatomine genomic population analysis. **(1)** gDNA is extracted from heads, legs and thorax of triatomine bugs. **(2)** After that, gDNA is processed using the 2b-RAD protocol [38] and **(3)** libraries are sequenced on Illumina instruments. **(4)** Once the data is delivered, it is trimmed and filtered before **(5)** used in genotyping software such as STACKS [58]. **(6)** Then, genotypes are exported from the cloud (MySQL repository) and filtered if large amount of missing data is present. **(7)** Finally, the polymorphic loci of interest are exported in conventional file formats for population genomic analysis. See Table 1 for an overview of the technique and for particular recommendations.

A salt extraction protocol modified from Aljanabi and Martinez [47] was used to extract total gDNA from *R*. *ecuadoriensis* heads, legs and thoraxes (hindgut excluded). The modified protocol involved an additional overnight chitinase digestion step, as well as one overnight 75% ethanol wash to ensure purity (Table 1 and Fig 2). gDNA concentrations and purity ratios assessments were obtained by using NanoDrop ND-1000 Spectrophotometer (NanoDrop Technologies, Inc.). Integrity of the extracted DNA was evaluated by agarose electrophoresis and highly fragmented samples were excluded from subsequent analysis.

**Table 1:** Overview of 2b-RAD genotyping: key considerations and further reading.

**1. gDNA extraction.**	Before the protocol starts, gDNA is extracted preferably from only crushed wings, thorax, legs, and head of triatomine bugs (or the vector of interest) to reduce/prevent contamination with gut bacteria or symbiotic fungi. It is crucial at this stage that very pure, high molecular weight, RNA-free gDNA at a concentration above 25 ng/μl is obtained for optimal restriction enzyme digestion [27].
**2. Library preparation.**	Once gDNA is obtained, the protocol is carried out in 3 consecutive steps: enzyme digestion, adaptor ligation, and amplification [38]; these differ for each RADseq variant (see [35–40,59] a detailed review). In the 2b-RAD protocol, a main consideration is to choose between a set of IIB-REases based on their performance *in silico* or during pilot experiments. Another feature of this protocol is its flexibility in using custom-made adaptors (oligonucleotide sequences in Wang *et al*. [38]) for controlling marker density depending on the type of study (e.g., hundreds to thousands of markers for genome-wide association and lower densities for mapping studies [38,40]).
**3. Sequencing.**	After library preparation, 2b-RAD tags are ready to be sequenced on Illumina platforms which provide a range of sequencing lengths (50–300 bp) along with other options such as single (forward) or paired-end sequencing (forward and reverse reads) [40]. Deciding which Illumina mode (NextSeq, MiSeq, HiSeq and TruSeq) to use may depend largely on project budget, as well as on the read depth and marker yield requirements of the study.
**4. Raw data cleaning.**	Before genotyping, 2b-RAD raw reads enter a quality control check using different software, such as FastQC, in which a per base score above 28 is expected (see [50] manual). Then, raw reads are cleaned by removing adaptors (trimming) and keeping (filtering) quality reads with the expected forward and reverse IIB-REase-specific recognition site. Finally, given that STACKS pipeline needs all reads in the same direction, reads with reverse recognition sites need to be forwarded (forwarding). Trimming, filtering and forwarding processes can be custom-coded in Python and/or PERL scripts, or alternatively, the PROCESS_RADTAGS module of STACKS can be used. This pre-genotyping cleaning assures that PCR and sequencing errors will not interfere with the next step.
**5. Genotype calling.**	Assembly of loci, either *de novo* or by alignment to a reference genome, can be performed using RADseq data handling software such as STACKS [58], UNEAK [60], PyRAD [61] or AftrRAD [62] among other pipelines. There is no rule of thumb when setting SNP calling parameters, and sensitivity analysis are always recommended to target the best values (see Methods). Perhaps, one should aim for detecting the highest number of SNPs while keeping a low error rate, which in STACKS is controlled mainly by the -m, -M, and -n parameters. Moreover, the—bound and—alpha parameters deal with true heterozygote/homozygote calling errors at a determinate locus.
**6. Genotype filtering.**	Before the run ends, the data is stored in MySQL database, which can be accessed via the STACKS EXPORT_SQL.PL utility and downloaded in a compact format (TSV or XLS). During the download, the user can specify different filters such as loci with a determinate number of SNPs, alleles per locus and percentage of sharing among samples. At this stage, additional filtering is recommended to remove loci or samples with a large amount of missing data and set a threshold for the polymorphic sharing by a fixed number of samples.
**7. Analysis.**	Finally, the previously identified loci can be exported in a standard output format, such as GENEPOP or STRUCTURE, using the STACKS POPULATIONS core program. From this stage, the biological information is ready to use for further conventional population genetics analysis (e.g., AMOVA, pairwise F_ST_, principal components and coordinates, and Bayesian clustering) or more recent approaches such as landscape genetics [63,64].

Almost a decade after the first RADseq [34,35] publication, RADseq markers are increasingly used in ecological, evolutionary and conservation genomic studies in non-model organisms [40] and more recently paving the way into epidemiology research. Such a recent but foremost extensive NGS technique cannot be fully covered in this work. Instead, we provide an overview of the steps and key considerations for setting up a 2b-RAD study. Moreover, further literature is indicated throughout each step to elaborate the technique.

### Type IIB restriction enzymes selection by *in silico* digestion

Initial selection of potential IIB-REases for our 2b-RAD protocol involved an *in silico* digestion of the *R*. *prolixus* genome, which is available from Genbank (accession code: KQ034056.1). For this purpose, 7 REases (AlfI, CspCI, BsaXI, SbfI, EcoRI, BcgI and KpnI) were screened. Three IIB-REases, namely AlfI, BcgI and CspCI were chosen based on the total number of restriction fragments produced *in silico* for the draft *R*. *prolixus* genome (www.vectorbase.org), financial resources, known efficiency in previous studies [31–33,38] and authors’ previous experience working with those enzymes [48]. We expected REases with abundant *in silico* restriction sites to show larger coverage variability among samples, at lower read depths. On the contrary, REases with less abundant restriction sites *in silico* could provide more exploitable markers at lower read depths.

### 2b-RAD library preparation and Illumina sequencing

Libraries were prepared using the 2b-RAD protocol proposed by Wang *et al*. [38] (Table 1 and Fig 2). Reaction mix and PCR conditions varied (S2 Table) depending on which IIB-REase was used. First, approximately 100–400 ng of high-quality gDNA from each sample was digested separately by each IIB-REase, producing IIB-REase-specific, uniform length fragments (32 bp, 35 bp and 33 bp for AlfI, BcgI and CspCI, respectively) with random overhangs. To confirm that the restriction reaction took place appropriately, equal amounts of digested DNA (dDNA) and gDNA from the same sample were visualized on a 1% agarose gel. Subsequently, the dDNA of each sample was ligated to a pair of partially double-stranded adaptors with compatible and fully degenerated overhangs (5’NNN3’). Finally, the obtained 2b-RAD tags were amplified to introduce a sample-specific 7bp barcode and the Illumina NGS annealing sites using two different pairs of sequencing primers. A 1.8% agarose gel electrophoresis of the PCR products was performed to verify the presence of the expected 150 bp target band (fragment, barcodes and adaptors included). In order to ensure an approximately equimolar contribution of each sample to the library, the exact amount of each PCR product was measured from the intensity of the target band in a digital image of the 1.8% agarose gel. We prepared three libraries in total, one for each IIB-REase, according to the relative concentration of each sample. The purification of the libraries from high-molecular weight fragments and primer-dimers was achieved first by removing the target band on agarose gel from each sample among the three libraries and eluting them in water overnight, followed by DNA capture with magnetic beads (SPRIselect Beckman Coulter) based on the Solid-Phase Reversible Immobilization method [49]. The DNA concentration in the purified libraries was quantified with a Qubit Fluorometer (Invitrogen) and the libraries were assembled in one single pool according to their relative concentrations. The library pool was sequenced on MiSeq (Illumina, San Diego, CA, USA) with a single 1x50 bp setup using ‘Version2’ chemistry at the Science for Life Laboratory (SciLifeLab, Stockholm, Sweden), which also implemented the reads demultiplexing and quality-filtering (Table 1 and Fig 2). Raw sequencing data has been uploaded to the Dryad Digital Repository (10.5061/dryad.02bf1).

### *In silico* assay to determine read depth vs locus recovery

The quality of demultiplexed and quality-filtered raw reads was verified by using FastQC software [50]. Subsequently, custom-made Python scripts were used for trimming the adaptors and then filtering the reads on the IIB-REase-specific recognition site (Table 1 and Fig 2). For each of the three libraries (AlfI, BcgI and CspCI) we sought to determine the relationship between sequencing effort (number of reads) and the total yield of polymorphic loci (set at up to two SNPs per locus). Therefore, we subsampled the total number of reads for each library in each individual using the fasta-subsample package from MEME SUITE [51] portal. This script randomly subsampled 25%, 50%, and 75% of total reads in triplicate to assess variability. This process resulted in 10 datasets per IIB-REase library: nine representing the three subsampling repetitions of the fixed percentages and only one from the total (100%) reads.

To estimate the polymorphic loci growth rate among the three IIB-REases, a nonlinear least square fitting (NLS) approach [52,53] was used with the R software [54] package NLS [55]. Specifically, NLS algorithm fits to the data by approximating a nonlinear function to a linear one, applying an iterative process to calculate the optimal parameter values for the growth rate [52,53,56]. Different built-in NLS models were tested in order to find the best fit to our data. These models were represented each with a different version of the Power-law equation [57]:

$$Y=aX^{b}$$ (1)

Here, *Y* is the expected number of polymorphic loci at reads yield *X*; *a* is the estimate starting amount of *Y* when *X* is close to 0; *b* is the estimate of the relative change of *Y* in relation to a unit change in *X* (slope). A detailed description of the equations used for each dataset is provided in S3 Table.

### Genotype calling and filtering

All datasets created were analyzed separately using STACKS software version 1.42 [58], in which *in silico* assembly of loci and individual genotyping was performed by running the DENOVO_MAP.PL pipeline (Table 1 and Fig 2). STACKS algorithm, first, reconstructs stacks (alleles) from exactly matching reads of each sample (-m). These stacks are then either merged with others to form a single polymorphic locus or kept as separate monomorphic loci depending on the number of nucleotide mismatches (-M). Stacks with repetitive sequences are removed from the pipeline. Finally, each sample information is stored in a catalog (stored in the MySQL repository) containing the consensus (-n) of all loci and alleles in the entire population (See [58] tutorials).

Due to the failure of the protocol in one of the samples from the AlfI library (likely as a result of low gDNA quality), we decided to discard this sample from the other two datasets to avoid biased results in the *de novo* assembly. After several parameter adjustments, we set the minimum number of identical raw reads necessary to create a stack (-m) to 5. We kept the number of mismatches allowed between loci when building a locus in a single individual (-M) and when comparing across all individuals to build the population catalogue (-n) at default values. The bounded SNP calling model for identifying a SNP and estimating the sequencing error rate for calling at that SNP (—bound) ranged from 0 to 0.05. Finally, the significance level required to call a heterozygote or homozygote (—alpha) was set to 0.01. The EXPORT_SQL.PL utility was used to export loci shared by at least the 80% and the 90% of samples with the same polymorphism level (loci with up to 2 SNPs) from the MySQL database for all datasets analyzed in STACKS for each IIB-REase (Table 1 and Fig 2).

### Population genomic analysis

Although both total number of samples (N = 19) and sample size per community (N = 4–5) were low, we conducted pilot explorations of the population structure of *R*. *ecuadoriensis* in the study area. We retained polymorphic loci shared by at least 90% of the samples, characterized by the presence of 1 and 2 SNPs and with a minor allele frequency of 0.01. We performed preliminary genomic analysis using two different datasets: i) one dataset contained 361 polymorphic loci obtained from 18 samples processed with the BcgI IIB-REase (one sample was excluded from the analysis due to the high level of missing data) and ii) the second dataset contained 1225 polymorphic loci obtained from 19 samples processed with the CspCI IIB-REase. The number of markers obtained for the AlfI dataset derived from digestion with AlfI was too low to be used for the preliminary assessment of genomic structure of this particular sample. During the genotype calling, it is possible for more than one SNP to appear within the same region. When two SNPs were recovered at a single locus, a conservative approach was used to retain the first SNP for analysis, thereby excluding tightly linked SNP variation.

ARLEQUIN version 3.5 [65] was used to calculate non-hierarchical analysis of molecular variance [AMOVA; 66]. To deal with missing data, the locus-by-locus option was set. Bayesian clustering implemented in STRUCTURE 2.3.4 [67] was conducted to investigate the most likely number of clusters of genetically related individuals excluding the locality origin (model LOPRIORI). After several trials, a burn-in of 300 000 followed by 3 million runs for K = 1 to K = 4 and 5 iterations per each K value was set; admixture model and correlated allelic frequencies were assumed. The most probable number of clusters was identified from delta K, implemented online with STRUCTURE HARVESTER [68]. Then, in order to confirm our polymorphic loci was *Rhodnius sp*.-related, we also aligned the total polymorphic loci shared by at least the 90% of samples obtained from BcgI and CspCI datasets to the reference *R*. *prolixus* genome using BOWTIE 1 [69]. The highest alignment score (*—best*) was chosen and no more than 3 mismatches (*-v*) were allowed.

## Results

### gDNA extraction and *in silico* digestion

The extraction method allowed us to obtain RNA-free genomic DNA from all twenty samples with an average DNA concentration (ng/μL) of 62.77 ± 33.75 (s.d.) with average DNA purity ratios of 1.81 ± 0.05 (s.d.) and 1.81 ± 0.62 (s.d.) for absorbance at 280/260 and at 260/230, respectively (see S1 Table for detailed information). The *in silico* digestion on *R*. *prolixus* genome sequence by AlfI, BcgI and CspCI IIB-REases produced 204895, 103268 and 69984 putative cut sites, respectively.

### 2b-RAD protocol

**Fig 3:**
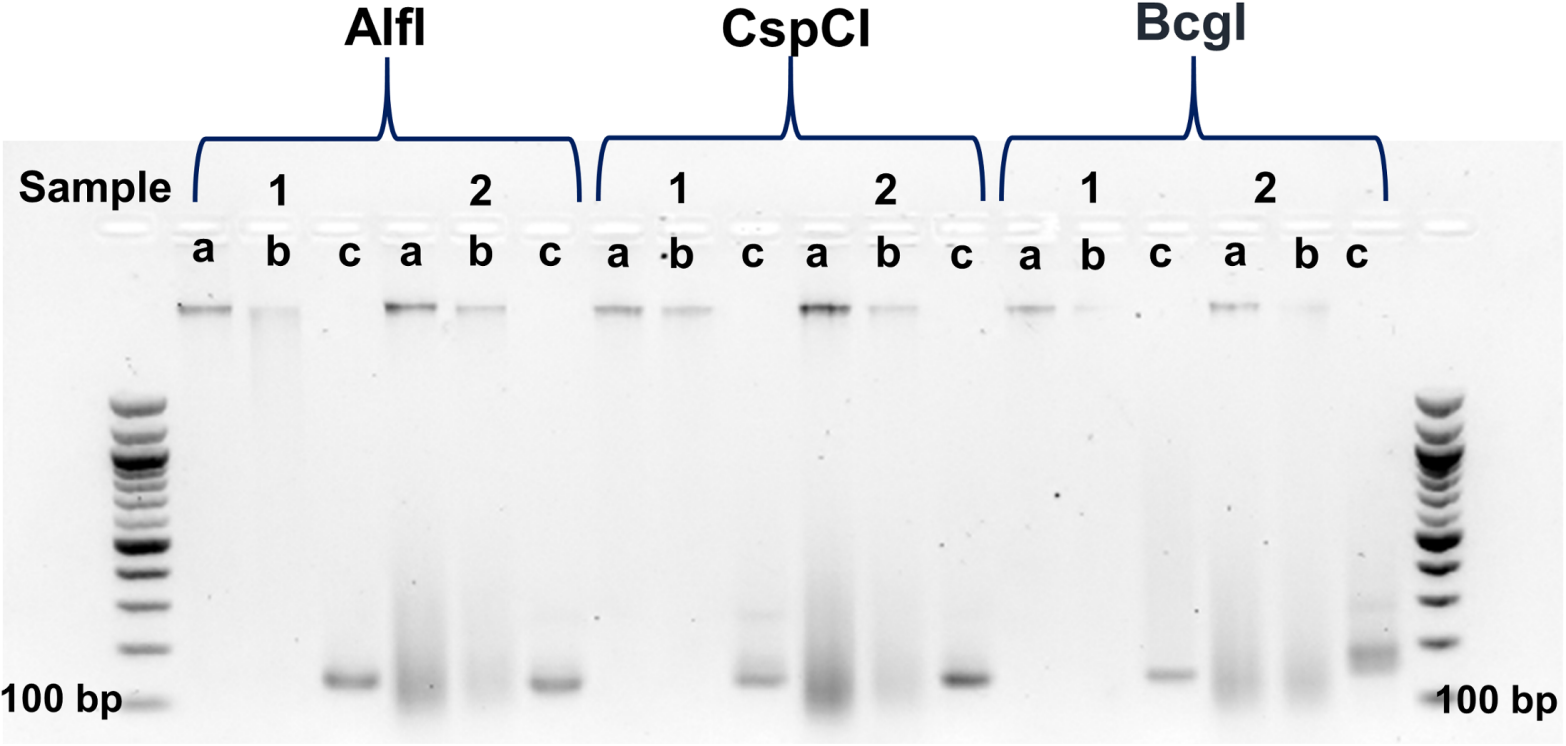
Example of two samples processed with 2b-RAD protocol. 1.8% agarose gel electrophoresis showing gDNA (a), digested DNA (b) and PCR product (c) in 2 samples for each of the 3 IIB-REases (AlfI, CspCI and BcgI).

The 2b-RAD experimental approach used in this study was effective for *R*. *ecuadoriensis* gDNA samples using any of the three IIB-REases (Fig 3), except for one sample (ID: CQ12, see S1 Table) digested by AlfI (CQ12 was thus not included in the pool for sequencing). A 2b-RAD pool of fifty-nine samples was established from nineteen samples digested by AlfI, twenty by BcgI, and twenty by CspCI IIB-REases.

### Sequencing data filtering and *de novo* analysis

**Fig 4:**
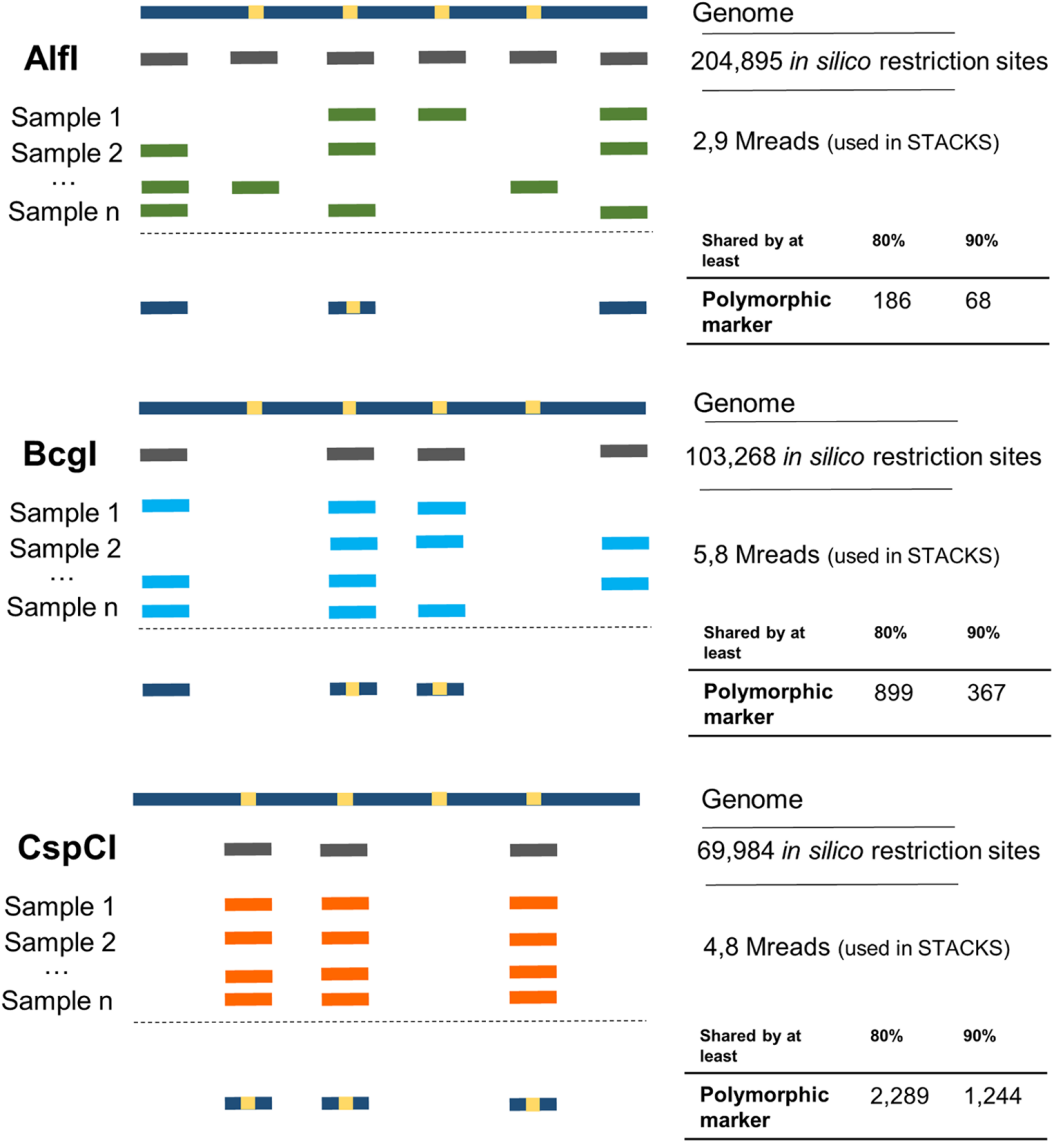
Comparison of read depth and marker identification in *R*. *ecuadoriensis* using 3 different Type IIB restriction enzymes. In line with *in silico* predictions, AlfI, an abundant *in silico* cutter did not produce enough molecular markers as compared to BcgI and CspCI, less abundant *in silico* cutters. In the diagram, enzymes with abundant *in silico* restriction sites (dark gray rectangles) within the genome (dark blue solid line with yellow squares or SNPs) are more likely to produce fragments (light blue, green and orange rectangles) at different locations among samples during a random experiment. This may yield insufficient read depth and thus compromise polymorphic marker discovery (dark blue rectangles with a yellow square).

The Illumina NGS yielded a total of 14.8 million de-multiplexed and quality-filtered reads, approximately 3, 6.2 and 5.6 million reads for AlfI, BcgI, and CspCI, respectively. FastQC analysis showed high per-base quality scores (> 32) for the reads of all samples processed with each of the three IIB-REases. After trimming the adaptors and filtering the IIB-REase-specific recognition site, 2.9, 5.8 and 4.8 million reads for AlfI, BcgI, and CspCI (respectively) were retained (Fig 4). The average trimmed Mreads per sample for each IIB-REase was 0.15 ± 0.06, 0.30 ± 0.04 and 0.25 ± 0.07. The number of reads subsampled and the total polymorphic loci for each IIB-REase are reported in Table 2. STACKS reference genome free runs assembled and identified a catalogue of loci from each of the datasets. The EXPORT_SQL.PL script was used to extract two datasets which included all the polymorphic loci with up to 2 SNPs shared by at least 80% and 90% of samples from each of the set percentages (25%, 50%, 75%, 100%) among the three replicates. We found only minor variation in the number of polymorphic loci called for each of the three subsampling replicates in all IIB-REase libraries. The average number of exported polymorphic loci obtained among replicates and from the total number of reads for each IIB-REase is reported in Table 2.

**Table 2:** Relationship between number of reads and polymorphic loci obtained from STACKS analysis.

IIB-REases	% Subsampled and total reads	Reads (Mreads)	Polymorphic Loci– 1 and 2 SNPs	
			90% sharing	80% sharing
AlfI	25	0.7	28.7 ± 2.5	47 ± 3
	50	1.4	51 ± 2	75.3 ± 4
	75	2.2	57.3 ± 3.1	99 ± 1.7
	100	2.9	68	186
BcgI	25	1.5	50 ± 5.2	78.3 ± 3.1
	50	2.9	100.7 ± 6.4	149 ± 6.1
	75	4.4	162 ± 6.2	331 ± 10.4
	100	5.8	367	899
CspCI	25	1.2	46.3 ± 3.8	65 ± 6.9
	50	2.4	81.7 ± 2.1	154.3 ± 5.9
	75	3.6	341 ± 10.6	995 ± 23.4
	100	4.8	1244	2289

Mean values provided ± the standard error.

**Fig 5:**
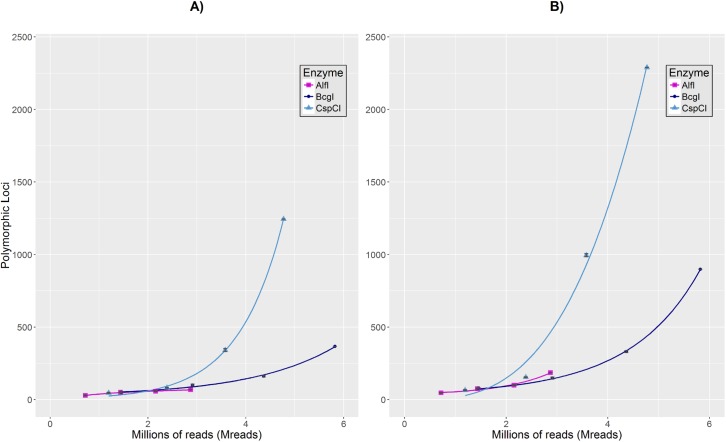
Relationship between the number of reads and polymorphic loci obtained by each Type IIB restriction enzyme. Lines show the comparison of the relationship between the increased number of reads obtained by AlfI (Magenta square), BcgI (Dark blue point) and CspCI (Light blue triangle) IIB-REases, and increasing numbers of polymorphic loci discovered after STACKS analysis. Different read abundances were obtained by randomly subsampling the dataset of each enzyme, and analyzing these in STACKS separately as independent datasets. In the figure, **A)** shows polymorphic loci with up to 2 SNPs shared by at least 90% of samples and best fit logarithmic (Magenta), geometric (Dark blue) and exponential (Light blue) growth curves. **B)** shows polymorphic loci with up to 2 SNPs shared by at least 80% of samples and best fit geometric (Magenta and Dark blue) and Power-law (Light blue) growth curves.

We observed growth in the number of loci recovered as we increased the read depth for all enzymes (Fig 5). However, while increasing read depth led to corresponding moderate and minor gains in locus number for BcgI and AlfI, respectively, for CspCI this number of loci is highlighted by a greater exponential growth in comparison to the other REases. Our results of best fit model analysis and estimated parameters (S3 Table) for each REase dataset were obtained by assessing different NLS models residual standard error, parameter significant p-values, number of iterations to convergence, the correlation between *y* and predicted values, and Akaike Information Criterion (AIC). In the first dataset (Fig 5A), we found that logarithmic (*y*∼*a*+*b*ln(*x*)), geometric (*y*∼*ax*^*bx*^) and exponential (*y*∼*ae*(*bx*)) NLS equations best fit to the AlfI, BcgI and CspCI datasets, respectively, allowing the estimation of growth rate parameters α and *b* (S3 Table). As for the second dataset (Fig 5B), geometric (*y*∼*ax*^*bx*^) and Power-law (*y*∼*ax*^*b*^) equations converged the best fit and parameters estimation for AlfI and BcgI, and CspCI, respectively (S3 Table). Detailed statistical analysis is provided in S1 Code.

### Preliminary population genomics analysis

**Fig 6:**
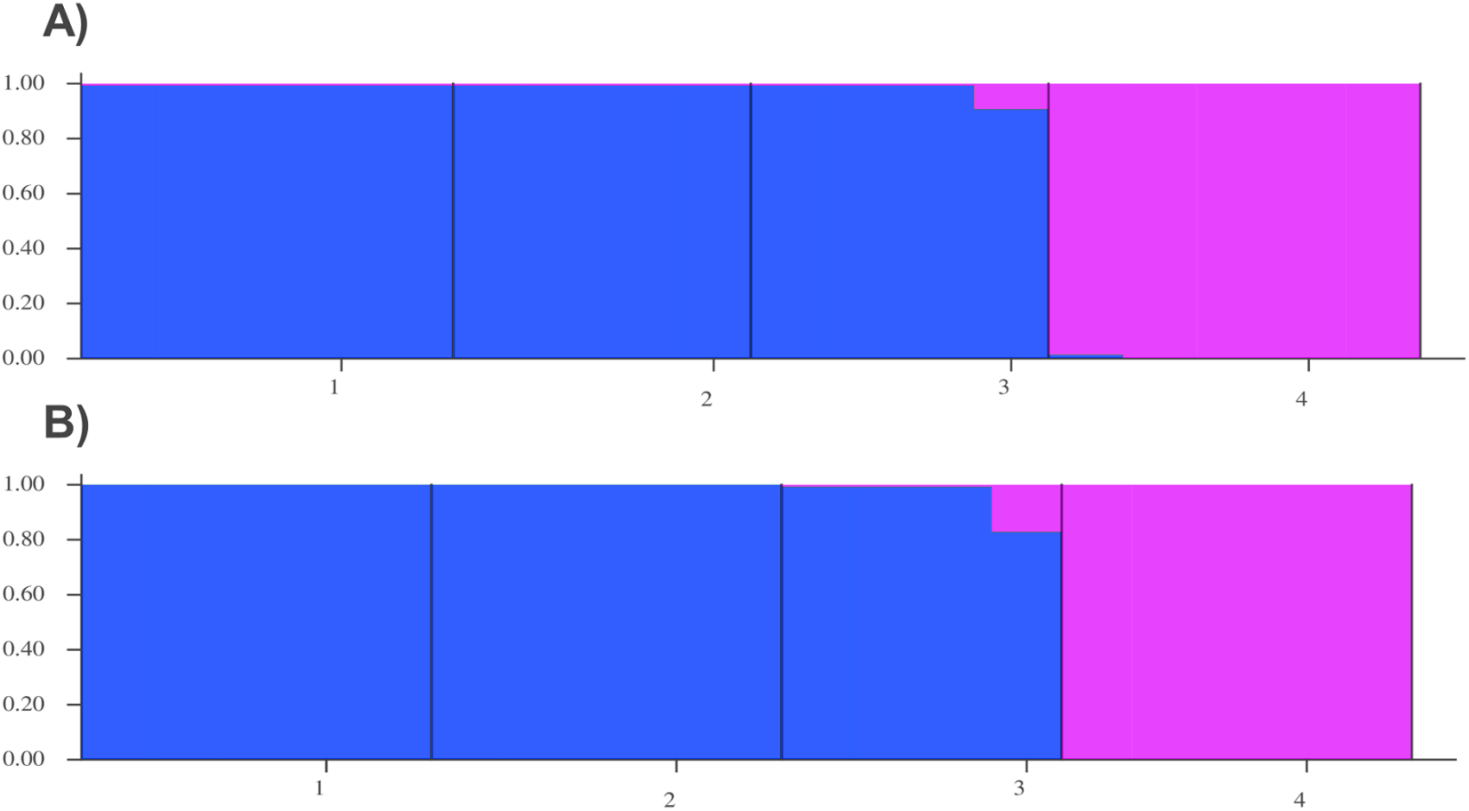
Genetic clusters (K = 2) assigned by STRUCTURE. The blue columns (1—CE, 2—EX and 3—CQ) indicate the samples from Loja, and the purple column (4—BJ) indicate the samples from Manabí. **A)** BcgI and **B)** CspCI datasets, respectively.

The non-hierarchical AMOVA carried out on all four community samples for both datasets (BcgI and CspCI) detected a strong signal of genetic structuring across the study area, with highly significant (P< 0.0001) global F_ST_ values of 0.20452 (BcgI) and 0.39327 (CspCI). The most likely number of genetic clusters (K) identified by STRUCTURE was 2 for both datasets: on one side, the 3 samples from Loja region (CE, EX, CQ) were grouped together, and on the other, the sample from Manabí (BJ) was considered as a distinct cluster (Fig 6). The alignment to the *R*. *prolixus* reference genome resulted in a 42% and 31% of polymorphic loci aligned for BcgI and CspCI, respectively, likely due to genomic variability between the *R*. *ecuadoriensis* and the available *R*. *prolixus* reference genome as well as the difficulty in mapping short reads.

## Discussion

Our data demonstrate the power of 2b-RAD as a valid genotyping approach that can be applied to Chagas disease vectors for which either no reference genome exists or, as in our case, a reference genome exists for a species within the same genus. Our data broadly support the assertion of Wang *et al*. [38] that the 2b-RAD approach provides a simple, cost-effective and robust means of generating genome wide SNP data for non-model organisms. In our experiment, library preparation and sequencing was completed within a month and the cost per sample was approximately $18 USD (library preparation and sequencing cost), as compared to $30 USD per sample in other RADseq methods [39]. In fact, costs and technical complexity are two of the key factors when considering different RADseq protocols for a particular genomic study [59]. Moreover, laboratories/research groups deciding between “going RAD” or “keeping it classic” in terms of genotyping should assess whether a certain marker type addresses the research question at hand and fits their current and future research ambitions along with project budget. A total project/per sample cost analysis study showed that the cost of genotyping using microsatellite loci ($17.58 for 24 loci in four multiplexes) was less expensive compared to SNPs ($39.35 for 288 pooled samples and using a ddRAD-seq protocol [37]). However, it was assumed that a set of 16–24 microsatellite loci and species-specific primers already existed [70], somewhat unrealistic for some non-model organisms in which microsatellite primer development and validation should still be carried out and be considered within the project costs. After a literature search, the authors also pointed out that when studies genotyped microsatellite and SNPs in the same samples, the latter provided higher accuracy and/or precision for parameter estimation.

### Type IIB restriction enzymes performance

In our study, we have gone somewhat further than a proof-of-principle by evaluating the performance of three distinct Type IIB restriction enzymes, pre-screened *in silico* for their performance in terms of marker density against the *Rhodnius prolixus* genome [23]. Our methodological development aim was to test the predictability of the *in silico* cutter and to provide recommendations for suitable read depths, marker numbers and sample sizes for studies involving *Rhodnius sp*. vectors. We expected that an abundant *in silico* enzyme cutter would provide less usable molecular markers at lower read depths (Fig 4). It is important to highlight that, enzyme performance *in silico* in terms of number of restriction sites is not necessarily the same in an actual experiment due to genome size, nucleotide distribution, depth of coverage and GC composition [27,40,71]. Thus, a pilot experiment always offers valuable information on actual restriction enzyme performance.

Random re-sampling (rarefaction) of our datasets revealed distinct relationships between read depth and marker (polymorphic locus) number between the different enzymes, CspCI, BcgI and AlfI (Fig 5) broadly in line with predictions of the number of usable markers (Fig 4). As such, CspCI produced the largest amount of polymorphic markers regardless of read depth, evidencing its experimental performance for *R*. *ecuadoriensis* and likely for other *Rhodnius* sp. vectors. AlfI and BcgI, on the other hand, showed a marked tendency of deceleration for marker recovery as read depth increases. However, AlfI does show the initially steeper growth, in line with predictions that AlfI cut sites in the *R*. *prolixus* genome are more abundant (AlfI = 204895 sites, BcgI = 103268 sites and CspCI = 69984 sites). Additionally, we were able to fit nonlinear regression models to the data and estimate growth rate parameters for each enzyme (Fig 5). Although the model function varies per enzyme and dataset, all of them follow an exponential growth pattern which is more evident in CspCI datasets. The model function applied to the second CspCI dataset (Fig 5B) did not entirely fit the data; however, it constitutes the best fit compared to generalized linear models or more complex NLS fitting functions. Fitting NLS models to fewer data points for parameter estimation is challenging; however, based on our best-fit selection process we were confident that by substituting *x* for a determinate read depth we can obtain an estimate of polymorphic loci growth per restriction enzyme. Moreover, both parameters, *a* and *b*, are crucial for estimating the starting number of polymorphic loci and shape of the growth curve and understanding how the number of polymorphic loci changes as the number of reads increases. We hope this will be helpful to others planning similar studies.

At the read depth we achieved on one Illumina MiSeq single-ended run across 20 *R*. *ecuadoriensis* DNA samples, we generated 1244 markers for CspCI, 367 for BcgI and 68 for AlfI. Even the lowest of these values eclipses the size of marker panels currently in use to explore Triatomine population genetics [8,13–20]. However, to generate read depths to exploit the higher density IIB-REase cutters (e.g. AlfI, BcgI), a HiSeq approach might be more sensible. On the other hand, based on our data, CspCI can be expected to generate the best coverage and over a thousand polymorphic markers for approximately sixty vector samples on one MiSeq run. Interestingly, Graham *et al*. [72] assessed the impact of degraded gDNA in a modified double-digest-RAD protocol [37] on the MiSeq platform and found a significant correlation between DNA degradation, read quality reduction and loss. They also suggested that a higher throughput platform, HiSeq, and short fragment producer protocols, such as 2b-RAD, could help dealing with degraded gDNA and subsequent sequencing problems. As such, 2b-RAD might be an option for research teams with large and long-term stored triatomine bug collections, in which gDNA might already have started degradation processes. Based on our study, CspCI is the best candidate for generating enough usable markers, seconded by BcgI, and it is likely that a sequencing platform such as HiSeq can exploit a higher number of markers for both enzymes.

### Preliminary population genomic analysis

As well as ‘range finding’ for the application of 2b-RAD sequencing to triatomine populations, our second aim was to undertake preliminary population genomic analysis to explore genetic structuring in our study region. To this end, we focused on datasets generated with BcgI and CspCI since they presented higher numbers of polymorphic loci. An AMOVA indicated a significant proportion of variation was explained by between-population differences for both datasets. Moreover, we demonstrated the feasibility of our markers to distinguish structuring among populations in both BcgI and CspCI datasets. By using a Bayesian clustering framework our markers from both data sets detected two distinct clusters without previous location information, one of them was Bejuco, the clear geographic outlier with respect to Loja populations. Morphometric and genetic studies of *R*. *ecuadoriensis* in Ecuador would also predict a similar pattern of diversification [73,74]. However, inter-population diversification in Loja might be happening [74] at a rate undetectable by coarse test for isolation-by-distance and other conventional population analysis techniques. Our genomic information coupled with a landscape genetics/genomics framework could test whether landscape heterogeneity and environmental variables are driving such processes [64].

### Overcoming 2b-RAD pitfalls and study limitations

Earlier in the manuscript we presented the notion that, fewer steps, simplicity, cost-effectiveness, fragment size and strand bias absence are advantages of using a 2b-RAD protocol compared to other RADseq methods. Nevertheless, researchers must be aware of potential pitfalls and sources of bias accompanying all RADseq protocols, as well as most NGS-based methods. However, development of sophisticated analysis and more powerful software tools to deal with the types of issues produced by most NGS platforms is an active and evolving field of research [75]. During the initial steps of library preparation, degraded gDNA seems to have a greater impact on read quantity and quality in all other RADseq protocols than in 2b-RAD [72]. However, guidelines [27] for assessing gDNA quality should be implemented in all protocols. Another drawback in all RADseq methods is that polymorphism can occur at the restriction site. This so-called allele dropout (ADO) prevents enzymes from cutting at that location and thus precludes recovery of that SNP allele (null allele) [40,76]. ADO will have a direct impact in the estimation of allele frequencies and consequently in overestimation/underestimation of F-statistics as individual heterozygote at the null allele will be recognized as homozygote. However, filtering loci successfully genotyped among a high percentage of the samples can help to remediate the problem [40]. PCR duplicates arise in all RADseq protocols with a PCR step, and only identifiable in protocols with a random shearing digestion (original RADseq protocol [35,36]) as duplicate fragments are identified by having the same length. Another promising approach described by Andrews *et al*. [40] to identify PCR duplicates is to use degenerated base regions within sequencing adaptors to mark parent fragments. However, Puritz *et al*. [43] highlighted that, though untested, skewed allele frequencies by PCR artefacts have little effect in statistical bias within loci and thereby genotype calling errors. No less important are sequencing errors introduced in all Illumina instruments. Although several genotype-calling algorithms account for sequencing errors, a high depth sequencing coverage (≥ 20x) is always recommended. Finally, sequencing depth variability among loci could reduce genotyping accuracy for some less covered loci, thus allowing for fewer individuals to be multiplexed per sequencing lane, i.e., increasing cost per sample [40,59].

In our study, most of the above issues encountered in RADseq have been circumvented either during the library preparation or the raw data filtering steps. Nevertheless, our main challenge is the absence of a reference genome to map short reads in order to ensure that all markers do indeed belong to *R*. *ecuadoriensis* and not to microorganisms such as bacteria and fungi. Furthermore, it may be important to differentiate between mitochondrial and autosomal loci or sex-specific chromosomes that might have an effect in population divergence analysis. To overcome this difficulty, we adopted a stringent approach during raw data trimming and genotype calling. We focused analyses to loci shared by a high proportion of individuals and removed loci and samples with high amounts of missing data.

### Further applications

Landscape genetics/genomics is a powerful and relatively new approach to explore the underlying spatial processes that affect genetic diversity in biological organisms [63]. Next to isolation-by-distance, isolation-by-resistance is a common null hypothesis tested in landscape genetics when more complex ecological and environmental processes are thought to be at play. The landscape genetics framework and tools such as causal modelling and environmental association analysis have the potential [63,64, 77–79] to uncover whether the same is true for *R*. *ecuadoriensis* genetic structuring and dispersal in Ecuador. In our study, the main limitation to carry out a wide range of conventional between and within-population analysis was the sample size per population. Low sample size required our analyses to consider an extended area to resist exploration of processes at finer geographic scales.

The high-resolution genotyping approach we have developed in this study now paves the way for landscape genetic/genomics analysis in vector-parasite systems [64], with genuine potential insights for rational disease and entomological control. For example, landscape genetics approaches expanded our understanding of the natural and human-aided dispersal dynamics of the invasive Asian tiger mosquito, *Aedes albopictus* [80]. Similarly, insecticide resistance gene spread in *Anopheles sinensis* has been tracked in China using landscape genetics approaches, demonstrating multiple origins and the importance of long term agricultural insecticide use [81]. More widely, high resolution SNP datasets are increasingly used to explore the local and international spread of important disease vectors (e.g. *Aedes aegypti* [29,82]).

2b-RAD typing not only promises a potential applicability for population genetic studies but also for linkage and quantitative loci mapping given that marker density can be controlled using selective adaptors [38]. In fact, via its GENOTYPE pipeline, the STACKS package potentiates the construction of genetic maps from F_2_ or backcrosses of *R*. *ecuadoriensis* or other triatomine species.

In conclusion, the decreasing cost and increasingly simplicity of approaches to generate high resolution SNP data puts such tools increasingly in the hands of researchers in endemic countries working on non-model organisms that act as vectors of Neglected Tropical Diseases. An analytical framework to incorporate detailed spatial and environmental variation into genetic analyses is now in place to facilitate a better understanding of the biology and dispersal of disease vectors.

## Supporting information

S1 Table Detailed information of *R*. *ecuadoriensis* samples used in this study. (PDF)

S2 Table Reagents and 2b-RAD protocol used in this study. (PDF)

S3 Table Results of the best fit model selection for each Type IIB-REase dataset. (PDF)

S1 Code Nonlinear least squares (NLS) analysis. (PDF)
