# Supplementary material for: 2b-RAD genotyping for population genomic studies of Chagas disease vectors: Rhodnius ecuadoriensis in Ecuador
Source: PLoS Negl Trop Dis. 2017 Jul 19;11(7):e0005710. doi: 10.1371/journal.pntd.0005710 (PMC5536387; doi:10.1371/journal.pntd.0005710)
Supplement: S1 Table — (PDF) [file pntd.0005710.s001.pdf]

S1 Table. Detailed information of *R. ecuadoriensis* samples used in this study.

Sample site and sample ID	DNA concentration ng/µL	260/280 ratio	260/230 ratio	Latitude	Longitude	House ID	Habitat	Collection site	Collection date (DD/MM/YY)	Life stage	T. cruzi infection
Chaquizhca, Loja.											
CQ4	37.2	1.82	1.59	-4.231105	-79.594118	CQ306	Intra-domicile	Kitchen wall	Fcol.09JUL2010	F	Positive
CQ10	66.4	1.95	1.63	-4.231105	-79.594118	CQ306	Intra-domicile	Kitchen wall	Fcol.09JUL2010	M	n.a
CQ11	43.8	1.85	1.57	-4.231105	-79.594118	CQ306	Intra-domicile	Kitchen wall	Fcol.09JUL2011	F	Negative
CQ12	32.9	1.86	1.39	-4.234881	-79.578047	CQ406	Peri-domicile	Chicken nest	Fcol.09JUL2012	M	Negative
CQ13	114.9	1.88	1.6	-4.12424	-79.7059	TC306	Peri-domicile	Chicken nest	Fcol.09JUL2013	M	Positive
Coamine, Loja.											
CE7	48.1	1.86	4.1	-4.117625	-79.62101394	CE509	Intra-domicile	Bed	Fcol.30JUN2010	M	Negative
CE8	74.8	1.76	1.83	-4.129398	-79.60071004	CE305	Peri-domicile	Chicken nest	Fcol.30JUN2010	F	Negative
CE9	49.1	1.82	2.55	-4.116617	-79.6163567	CE505	Peri-domicile	Hen house	Fcol.30JUN2010	M	Positive
CE10	97.3	1.88	2.18	-4.116617	-79.6163567	CE505	Peri-domicile	Hen house	Fcol.30JUN2010	F	Positive
CE11	81.4	1.83	1.75	-4.116617	-79.6163567	CE505	Peri-domicile	Hen house	Fcol.30JUN2010	M	Negative
La Extensa, Loja.											
EX3	29.5	1.73	1.47	-4.043671	-79.359601	EX608	Peri-domicile	Chicken nest	F. col26JUN2006	F	n.a
EX8	28.5	1.65	1.25	-4.043671	-79.359601	EX608	Peri-domicile	Bed	F. col26JUN2006	F	n.a
EX9	32.5	1.76	2.15	-4.04413	-79.35964	EX803	Peri-domicile	Chicken nest	F. col8AUG2007	F	Negative
EX10	52.7	1.74	1.4	-4.04413	-79.35964	EX803	Peri-domicile	Chicken nest	F. col8AUG2007	F	Negative
EX11	150.9	1.87	1.5	-4.04413	-79.35964	EX803	Peri-domicile	Chicken nest	F. col8AUG2007	F	Negative

Bejuco, Manabí.											
BJ5	72.7	1.81	1.64	-0.9728	-80.3445	BJN55-1	Peri-domicile	Rat nest	F. col23JUN2007	M	n.a
BJ10	25.7	1.67	1.73	-0.9728	-80.3445	TBJ1940	Peri-domicile	Chicken nest	Fcol.07JUL2009	M	n.a
BJ11	71.1	1.8	1.52	-0.9728	-80.3445	TBJ1941	Peri-domicile	Chicken nest	Fcol.07JUL2009	M	n.a
BJ12	40.5	1.86	1.93	-0.9728	-80.3445	TBJ2009	Peri-domicile	Chicken nest	Fcol.13OCT2009	M	n.a
BJ13	105.4	1.8	1.48	-0.9728	-80.3445	TBJ2010	Peri-domicile	Chicken nest	Fcol.13OCT2009	M	n.a
