# Supplementary material for: 2b-RAD genotyping for population genomic studies of Chagas disease vectors: Rhodnius ecuadoriensis in Ecuador
Source: PLoS Negl Trop Dis. 2017 Jul 19;11(7):e0005710. doi: 10.1371/journal.pntd.0005710 (PMC5536387; doi:10.1371/journal.pntd.0005710)
Supplement: S2 Table — (PDF) [file pntd.0005710.s002.pdf]

S2 Table. Reagents and 2b-RAD protocol used in this study.

	1. Digestion	2. Ligation	3. Amplification
AlfI	**Reaction mix:**<br><br>**Reagent | 1x**<br><br>Buffer R 10X 0.6 µL<br>SAM 100 µM 0.6 µL<br>dd H_2O 0.05 µL<br>AlfI * 2 U/µL 0.75 µL<br><br>**Combine:**<br><br>2 µL Master mix + 4 µL genomic DNA<br><br>**Reaction profile:**<br><br>37 °C for 2 hrs<br>65 °C for 20 min<br>16 °C for ∞	**Reaction mix:**<br><br>**Reagent | 1x**<br><br>dd H_2O 9.5 µL<br>Buffer 2.5 µL<br>ATP** 10 µM 0.5 µL<br>Adaptor 2 2.5 µL<br>Adaptor 3 2.5 µL<br>T4 Ligase** 2.5 µL<br><br>**Combine:**<br><br>20 µL Master mix + 5 µL digested DNA<br><br>**Reaction profile:**<br><br>16 °C for 3 hrs<br>65 °C for 10 min<br>4 °C for overnight	**Reaction mix:**<br><br>**Reagent | 1x**<br><br>dd H_2O 14.7 µL<br>Buffer 10 µL<br>dNTP 2 mM 7.8 µL<br>Primer F 2.5 µL<br>pAMPF 1 µL<br>pAMPR 1 µL<br>Taq polymerase** 0.5 µL<br><br>**Combine:**<br><br>37.5 µL Master mix + 10 µL ligated DNA + 2.5 µL barcoded-primer<br><br>**Reaction profile:**<br><br>14 cycles:<br>98 °C for 5 sec<br>60 °C for 30 sec<br>72 °C for 5 sec<br><br>Then:<br><br>72 °C for 5 min<br>16 °C for ∞
BcgI	**Reaction mix:**<br><br>**Reagent | 1x**<br><br>Buffer 0.8 µL<br>SAM 100 µM 0.7 µL<br>BcgI ** 2 U/ µL 0.5 µL<br><br>**Combine:**<br><br>2 µL Master mix + 4 µL genomic DNA<br><br>**Reaction profile:**<br><br>37 °C for 1 hr<br>65 °C for 20 min<br>4 °C for 2 hrs	**Reaction mix:**<br><br>**Reagent | 1x**<br><br>dd H_2O 9.5 µL<br>Buffer 2.5 µL<br>ATP** 10 mM 0.5 µL<br>Adaptor 2 2.5 µL<br>Adaptor 3 2.5 µL<br>T4 Ligase** 2.5 µL<br><br>**Combine:**<br><br>20 µL Master mix + 5 µL digested DNA<br><br>**Reaction profile:**<br><br>16 °C for 2 hrs<br>65 °C for 10 min<br>4 °C for 2 hrs	**Reaction mix:**<br><br>**Reagent | 1x**<br><br>Same as *AlfI*'s reagent and quantities<br><br>**Combine:**<br><br>37.5 µL Master mix + 10 µL ligated DNA + 2.5 µL barcoded-primer<br><br>**Reaction profile:**<br><br>16 cycles:<br>98 °C for 5 sec<br>60 °C for 30 sec<br>72 °C for 5 sec<br><br>Then:<br><br>72 °C for 5 min<br>16 °C for ∞

<table>
<tr><td></td><td></td><td></td><td></td></tr>
<tr><td>CspCI</td><td>Reaction mix:

Reagent | 1x

NEB Buffer 0.6 µL
SAM 100 µM 1.2 µL
CspCI ** 1.5 U/µL 0.75 µL

Combine:

2 µL Master mix + 4 µL genomic DNA

Reaction profile:

37 °C for overnight
Refill samples with 0.2 µL CspCI after 2 hrs
65 °C for 10 min
16 °C for ∞</td><td>Reaction mix:

Reagent | 1x

dd H_2O 9.5 µL
Buffer 2.5 µL
ATP** 10 µM 0.5 µL
Adaptor 2 2.5 µL
Adaptor 3 2.5 µL
T4 Ligase** 2.5 µL

Combine:

20 µL Master mix + 5 µL digested DNA

Reaction profile:

16 °C for overnight
16 °C for 10 min
4 °C for ∞</td><td>Reaction mix:

Reagent | 1x

Same as AlfI's reagents and quantities

Combine:

37.5 µL Master mix + 10 µL ligated DNA + 2.5 µL barcoded-primer

Reaction profile:

16 cycles:
98 °C for 5 sec
60 °C for 30 sec
72 °C for 5 sec

Then:

72 °C for 5 min
16 °C for ∞</td></tr>
<tr><td></td><td colspan="3">Note: Adaptor 2 was made from oligonucleotide anti-ILL and 5ILL-NN; Adaptor 3 was made of oligonucleotide anti-ILL and 3ILL-NN from Wang et al. 2012. * Thermo Fisher Scientific; ** New England BioLabs inc.</td></tr>
</table>
