# Supplementary material for: 2b-RAD genotyping for population genomic studies of Chagas disease vectors: Rhodnius ecuadoriensis in Ecuador
Source: PLoS Negl Trop Dis. 2017 Jul 19;11(7):e0005710. doi: 10.1371/journal.pntd.0005710 (PMC5536387; doi:10.1371/journal.pntd.0005710)
Supplement: S3 Table — (PDF) [file pntd.0005710.s003.pdf]

S3 Table. Results of the best fit model selection for each Type IIB-REase dataset.

	NLS model equation	Residual standard error	Parameters and P-values	Iterations to convergence	Correlation y vs. predicted values	AIC
**Polymorphic loci with up to 2 SNPs shared by at least 90% of samples**						
AlfI	Logarithmic $y \sim a + b\ln(x)$	2.49, df=2	a = 38.5, p < 0.001<br>b = 27.4, p < 0.001	1	0.99	19.8
BcgI	Geometric $y \sim ax^{bx}$	11.08, df=2	a = 47.7, p < 0.01<br>b = 0.20, p < 0.001	13	0.99	31.8
CspCI	Exponential $y \sim ae(bx)$	16.82, df=2	a = 6.9, p < 0.01<br>b = 1.1, p < 0.001	9	0.99	35.2
**Polymorphic loci with up to 2 SNPs shared by at least 80% of samples**						
AlfI	Geometric $y \sim ax^{bx}$	7.87, df = 2	a = 54.4, p < 0.001<br>b = 0.4, p < 0.001	6	0.99	29.1
BcgI	Geometric $y \sim ax^{bx}$	6.39, df = 2	a = 64.8, p < 0.001<br>b = 0.26, p < 0.0001	13	0.99	27.4
CspCI	Pow er-law $y \sim ax^{b}$	91.3, df = 2	a = 16.7, p < 0.1<br>b = 3.2, p < 0.001	6	0.99	48.7
