# Supplementary material for: 2b-RAD genotyping for population genomic studies of Chagas disease vectors: Rhodnius ecuadoriensis in Ecuador
Source: PLoS Negl Trop Dis. 2017 Jul 19;11(7):e0005710. doi: 10.1371/journal.pntd.0005710 (PMC5536387; doi:10.1371/journal.pntd.0005710)
Supplement: S1 Code — (PDF) [file pntd.0005710.s004.pdf]

S1 Code. Nonlinear least squares (NLS) analysis.

Luis Enrique Hernandez

Tue May 30 13:36:59 2017

==

This code creates several models based on nonlinear regression (nonlinear least squares) and Local Polynomial Regression Fitting (LOESS).

These models aim to fit into data that describes the relation between the number of 2b-RAD sequenced reads needed to achieve a determinate polymorphic loci yield.

date: 30th May 2017

```
setwd("C:/Users/enrique/Desktop/PhD publication
submissions/PlosNTD_V09MAY2017_3rdSubmission/Fig5_plot")
```

===

Read the first data set - Polymorphic loci shared by 90% of total samples

```
# Load packages

data1 <- read.csv("2SNP90.csv")
attach(data1)
library("ggplot2")

## Warning: package 'ggplot2' was built under R version 3.2.5

library("ggthemes")
library("scales")
install.packages("extrafont", repos = "http://cran.us.r-project.org")

## Installing package into 'C:/Users/enrique/Documents/R/win-library/3.2'
## (as 'lib' is unspecified)

## Warning: unable to access index for repository http://cran.us.r-
project.org/src/contrib:
##   cannot open URL 'http://cran.us.r-project.org/src/contrib/PACKAGES'

## Warning: package 'extrafont' is not available (for R version 3.2.4)

## Warning: unable to access index for repository http://cran.us.r-
project.org/bin/windows/contrib/3.2:
```

```
##   cannot open URL 'http://cran.us.r-
project.org/bin/windows/contrib/3.2/PACKAGES'
```

```
library(extrafont)
```

```
## Warning: package 'extrafont' was built under R version 3.2.5
```

```
## Registering fonts with R
```

```
font_import(pattern="[A/a]rial")
```

```
## Importing fonts may take a few minutes, depending on the number of fonts
and the speed of the system.
## Continue? [y/n]
```

```
## Exiting.
```

```
require(ggplot2)
require(ggthemes)
require(scales)
require(extrafont)

#fonts()
#fonttable()
#loadfonts(device="win")
```

================================

Create data frames with summarized data from the 3 repetitions, in which 25%, 50%, 75% and 100% of the reads were randomly chosen to be analized in STACKS for SNP call.

```
# AlfI subset data set

MeanAlfI25 <- mean(subset(data1, Enzyme == "AlfI25")$Loci)
SdAlfI25 <- sd(subset(data1, Enzyme == "AlfI25")$Loci)
SeAlfI25 <- sd(subset(data1, Enzyme ==
"AlfI25")$Loci)/sqrt(length(subset(data1, Enzyme == "AlfI25")$Loci))

MeanAlfI50 <- mean(subset(data1, Enzyme == "AlfI50")$Loci)
SdAlfI50 <- sd(subset(data1, Enzyme == "AlfI50")$Loci)
SeAlfI50 <- sd(subset(data1, Enzyme ==
"AlfI50")$Loci)/sqrt(length(subset(data1, Enzyme == "AlfI50")$Loci))

MeanAlfI75 <- mean(subset(data1, Enzyme == "AlfI75")$Loci)
SdAlfI75 <- sd(subset(data1, Enzyme == "AlfI75")$Loci)
SeAlfI75 <- sd(subset(data1, Enzyme ==
"AlfI75")$Loci)/sqrt(length(subset(data1, Enzyme == "AlfI75")$Loci))

MeanAlfI100 <- mean(subset(data1, Enzyme == "AlfI100")$Loci)
SdAlfI100 <- sd(subset(data1, Enzyme == "AlfI100")$Loci)
SeAlfI100 <- sd(subset(data1, Enzyme ==
```

```
"AlfI100")$Loci)/sqrt(length(subset(data1, Enzyme == "AlfI100")$Loci))

# BcgI subset data set

MeanBcgI25 <- mean(subset(data1, Enzyme == "BcgI25")$Loci)
SdBcgI25 <- sd(subset(data1, Enzyme == "BcgI25")$Loci)
SeBcgI25 <- sd(subset(data1, Enzyme ==
"BcgI25")$Loci)/sqrt(length(subset(data1, Enzyme == "BcgI25")$Loci))

MeanBcgI50 <- mean(subset(data1, Enzyme == "BcgI50")$Loci)
SdBcgI50 <- sd(subset(data1, Enzyme == "BcgI50")$Loci)
SeBcgI50 <- sd(subset(data1, Enzyme ==
"BcgI50")$Loci)/sqrt(length(subset(data1, Enzyme == "BcgI50")$Loci))

MeanBcgI75 <- mean(subset(data1, Enzyme == "BcgI75")$Loci)
SdBcgI75 <- sd(subset(data1, Enzyme == "BcgI75")$Loci)
SeBcgI75 <- sd(subset(data1, Enzyme ==
"BcgI75")$Loci)/sqrt(length(subset(data1, Enzyme == "BcgI75")$Loci))

MeanBcgI100 <- mean(subset(data1, Enzyme == "BcgI100")$Loci)
SdBcgI100 <- sd(subset(data1, Enzyme == "BcgI100")$Loci)
SeBcgI100 <- sd(subset(data1, Enzyme ==
"BcgI100")$Loci)/sqrt(length(subset(data1, Enzyme == "BcgI100")$Loci))

# CspCI subset data set

MeanCspCI25 <- mean(subset(data1, Enzyme == "CspCI25")$Loci)
SdCspCI25 <- sd(subset(data1, Enzyme == "CspCI25")$Loci)
SeCspCI25 <- sd(subset(data1, Enzyme ==
"CspCI25")$Loci)/sqrt(length(subset(data1, Enzyme == "CspCI25")$Loci))

MeanCspCI50 <- mean(subset(data1, Enzyme == "CspCI50")$Loci)
SdCspCI50 <- sd(subset(data1, Enzyme == "CspCI50")$Loci)
SeCspCI50 <- sd(subset(data1, Enzyme ==
"CspCI50")$Loci)/sqrt(length(subset(data1, Enzyme == "CspCI50")$Loci))

MeanCspCI75 <- mean(subset(data1, Enzyme == "CspCI75")$Loci)
SdCspCI75 <- sd(subset(data1, Enzyme == "CspCI75")$Loci)
SeCspCI75 <- sd(subset(data1, Enzyme ==
"CspCI75")$Loci)/sqrt(length(subset(data1, Enzyme == "CspCI75")$Loci))

MeanCspCI100 <- mean(subset(data1, Enzyme == "CspCI100")$Loci)
SdCspCI100 <- sd(subset(data1, Enzyme == "CspCI100")$Loci)
SeCspCI100 <- sd(subset(data1, Enzyme ==
"CspCI100")$Loci)/sqrt(length(subset(data1, Enzyme == "CspCI100")$Loci))
```

```r
# Build a data frame with all the results from previous subsets.

Enzyme <- c("AlfI", "AlfI", "AlfI", "AlfI", "BcgI", "BcgI", "BcgI","BcgI",
"CspCI", "CspCI", "CspCI", "CspCI")
PolyLoci <- c(MeanAlfI25, MeanAlfI50, MeanAlfI75, MeanAlfI100, MeanBcgI25,
MeanBcgI50, MeanBcgI75, MeanBcgI100, MeanCspCI25, MeanCspCI50, MeanCspCI75,
MeanCspCI100)
Reads <- c(718061, 1436126, 2154183, 2872241, 1455376, 2910750, 4366118,
5821489, 1192825, 2385650, 3578472, 4771292)/1000000
SD <- c(SdAlfI25, SdAlfI50, SdAlfI75, SdAlfI100, SdBcgI25, SdBcgI50,
SdBcgI75, SdBcgI100, SdCspCI25, SdCspCI50, SdCspCI75, SdCspCI100)
SE <- c(SeAlfI25, SeAlfI75, SeAlfI50, SeAlfI100, SeBcgI25, SeBcgI50,
SeBcgI75, SeBcgI100, SeCspCI25, SeCspCI50, SeCspCI75, SeCspCI100)
IISNP_df <- data.frame(Reads, PolyLoci, SD, SE, Enzyme)

IISNP_df

##       Reads   PolyLoci        SD        SE Enzyme
## 1  0.718061   28.66667  2.516611 1.452966   AlfI
## 2  1.436126   51.00000  2.000000 1.763834   AlfI
## 3  2.154183   57.33333  3.055050 1.154701   AlfI
## 4  2.872241   68.00000  0.000000 0.000000   AlfI
## 5  1.455376   50.00000  5.196152 3.000000   BcgI
## 6  2.910750  100.66667  6.429101 3.711843   BcgI
## 7  4.366118  162.00000  6.244998 3.605551   BcgI
## 8  5.821489  367.00000  0.000000 0.000000   BcgI
## 9  1.192825   46.33333  3.785939 2.185813  CspCI
## 10 2.385650   81.66667  2.081666 1.201850  CspCI
## 11 3.578472  341.00000 10.583005 6.110101  CspCI
## 12 4.771292 1244.00000  0.000000 0.000000  CspCI
```

==

Subset from the II_SNP_df data frame information of each Enzyme to use in Build-in Nonlinear Regression models constructionm and LOESS. It will also be used for the in-figure equation annotation.

```r
Alfeq <- subset(IISNP_df, Enzyme=="AlfI")
Bcgeq <- subset(IISNP_df, Enzyme == "BcgI")
Cspeq <- subset(IISNP_df, Enzyme == "CspCI")
```

==

Nonlinear regression - nls models - statistical analysis

To calculate the parameters a and b for our nls models:

Simply build a "lm" model for each of the Enzyme data frames to calculate (more accurately) the slope and intercept which will be your a and b starting values parameters

for your nonlinear model. If your starting values, a and b, are very odd the "nls" algorithm won't converge and you will see an error message.

Parameter a is the approximate y value when x=0

Parameter b is the decay rate

Additionally, We added an ANOVA analysis to assess whether the model fit better with 1 or 2 parameters.

```
parmA <- lm(log(Alfeq$PolyLoci)~log(Alfeq$Reads))
anova(update(parmA, ~ . -1), parmA)

## Analysis of Variance Table
##
## Model 1: log(Alfeq$PolyLoci) ~ log(Alfeq$Reads) - 1
## Model 2: log(Alfeq$PolyLoci) ~ log(Alfeq$Reads)
##   Res.Df     RSS Df Sum of Sq      F    Pr(>F)
## 1      3 29.0316
## 2      2  0.0149  1    29.017 3895.6 0.0002566 ***
## ---
## Signif. codes:  0 '***' 0.001 '**' 0.01 '*' 0.05 '.' 0.1 ' ' 1

plot(parmA)
```

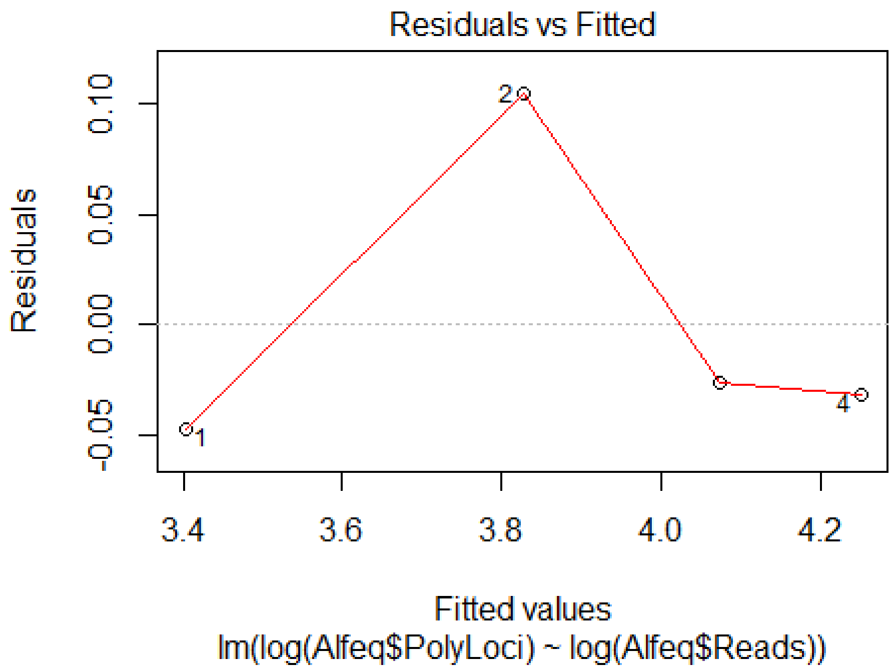
Residuals vs Fitted
Residuals
0.10
0.05
0.00
-0.05
3.4
3.6
3.8
4.0
4.2
Fitted values
lm(log(Alfeq$PolyLoci) ~ log(Alfeq$Reads))

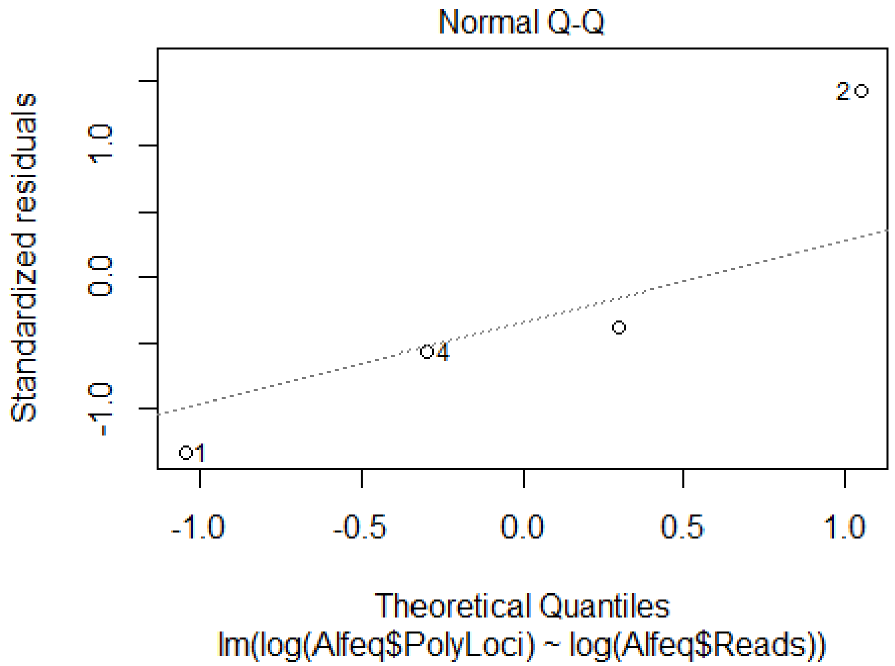
Normal Q-Q
Standardized residuals
1.0
0.0
-1.0
-1.0
-0.5
0.0
0.5
1.0
Theoretical Quantiles
lm(log(Alfeq$PolyLoci) ~ log(Alfeq$Reads))

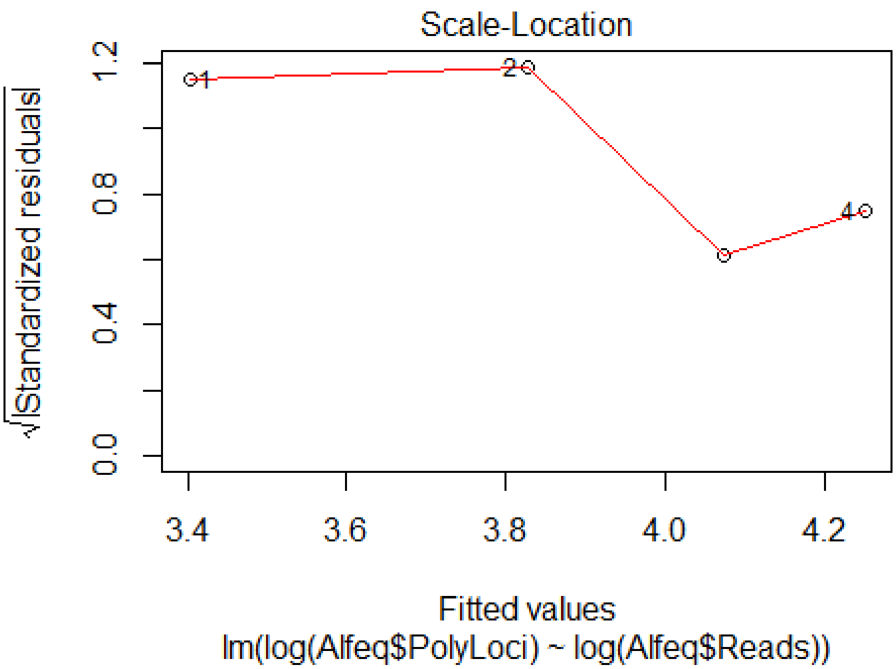

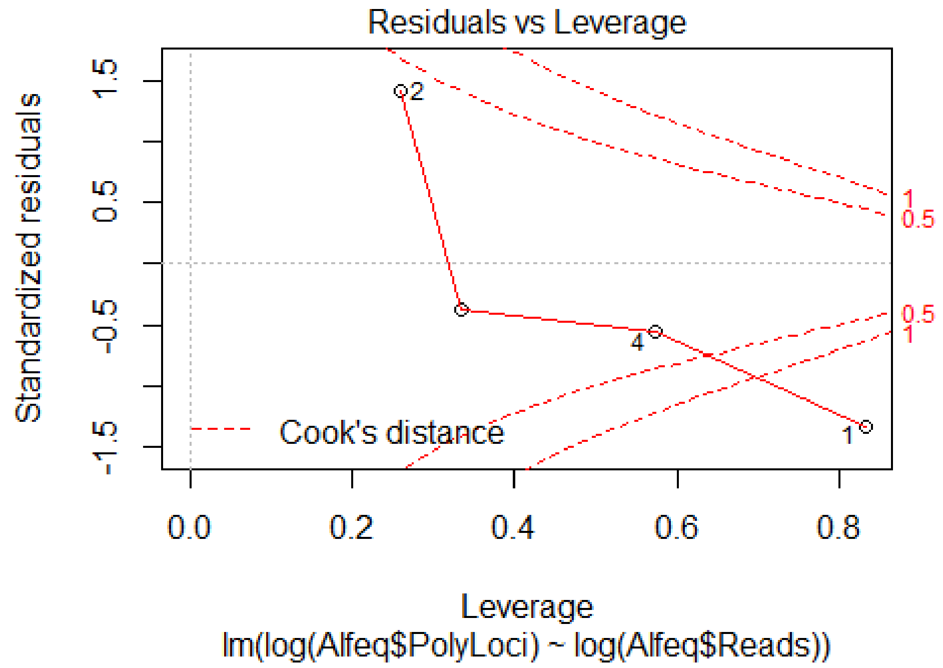


```
parmB <- lm(log(Bcgeq$PolyLoci)~log(Bcgeq$Reads))
anova(update(parmB, ~ . -1), parmB)
```

```
## Analysis of Variance Table
##
## Model 1: log(Bcgeq$PolyLoci) ~ log(Bcgeq$Reads) - 1
## Model 2: log(Bcgeq$PolyLoci) ~ log(Bcgeq$Reads)
##   Res.Df    RSS Df Sum of Sq      F   Pr(>F)
## 1      3 7.3108
## 2      2 0.1207  1    7.1901 119.19 0.008286 **
## ---
## Signif. codes:  0 '***' 0.001 '**' 0.01 '*' 0.05 '.' 0.1 ' ' 1

plot(parmB)
```

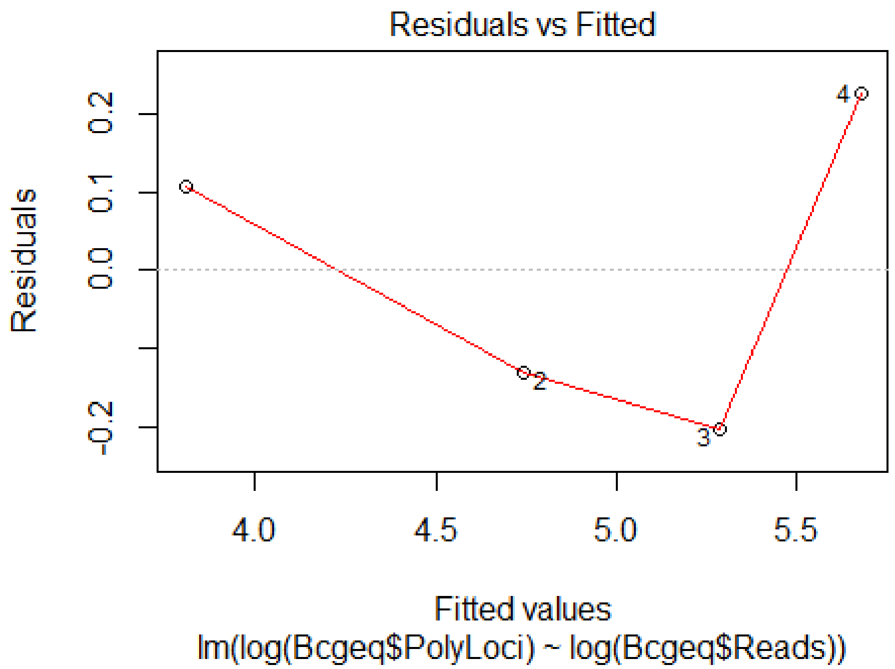

Residuals vs Fitted
Residuals
Fitted values
lm(log(Bcgeq$PolyLoci) ~ log(Bcgeq$Reads))

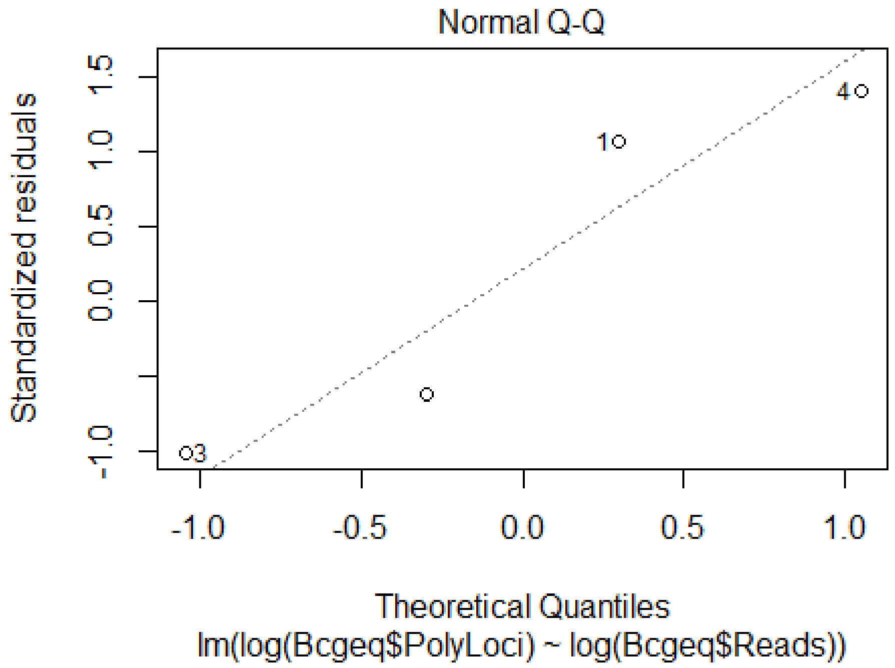

Normal Q-Q
Standardized residuals
Theoretical Quantiles
lm(log(Bcgeq$PolyLoci) ~ log(Bcgeq$Reads))

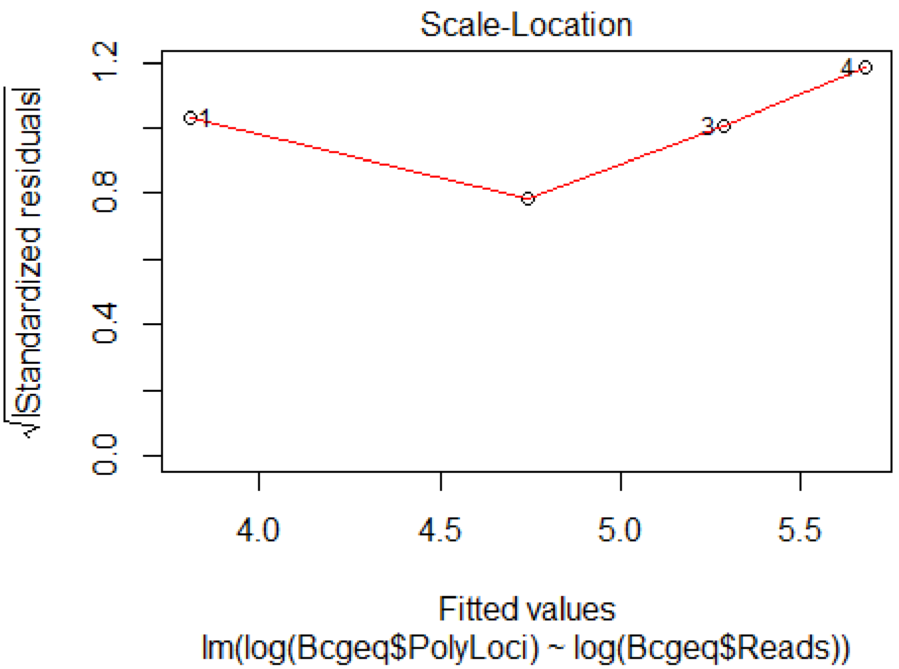

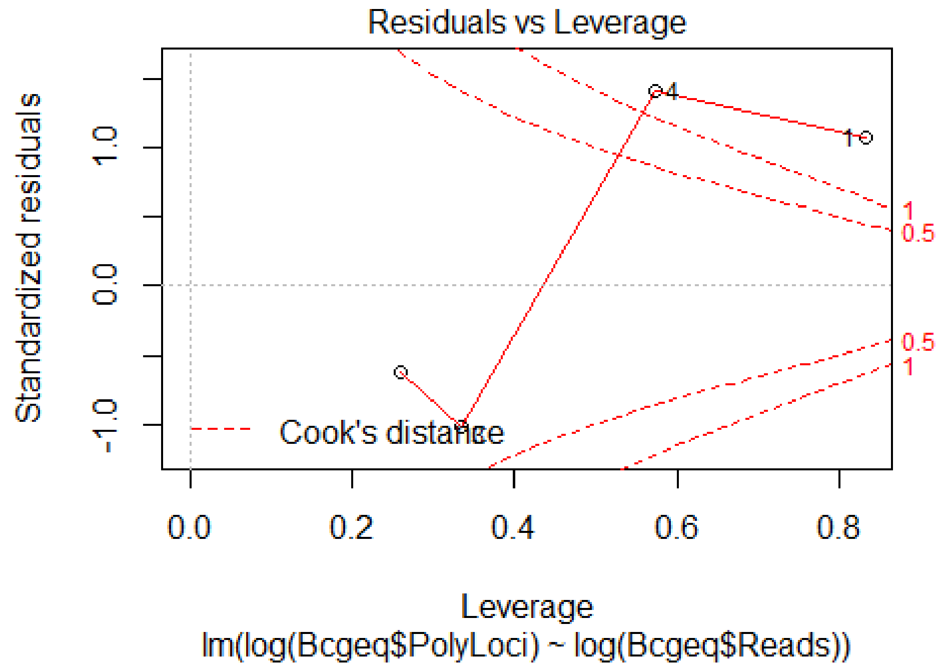


```
parmC <- lm(log(Cspeq$PolyLoci)~log(Cspeq$Reads))
anova(update(parmC, ~ . -1), parmC)
```

```
## Analysis of Variance Table
##
## Model 1: log(Cspeq$PolyLoci) ~ log(Cspeq$Reads) - 1
## Model 2: log(Cspeq$PolyLoci) ~ log(Cspeq$Reads)
##   Res.Df    RSS Df Sum of Sq      F  Pr(>F)
## 1      3 9.2022
## 2      2 0.8174  1    8.3848 20.516 0.04545 *
## ---
## Signif. codes:  0 '***' 0.001 '**' 0.01 '*' 0.05 '.' 0.1 ' ' 1

plot(parmC)
```

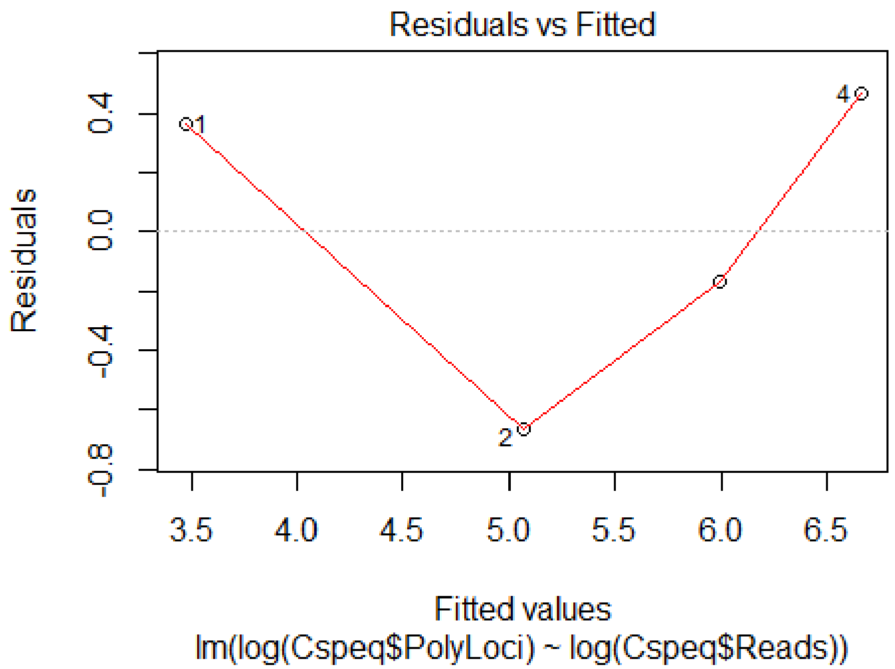

Residuals vs Fitted
Residuals
0.4
0.0
-0.4
-0.8
1
2
4
3.5
4.0
4.5
5.0
5.5
6.0
6.5
Fitted values
lm(log(Cspeq$PolyLoci) ~ log(Cspeq$Reads))

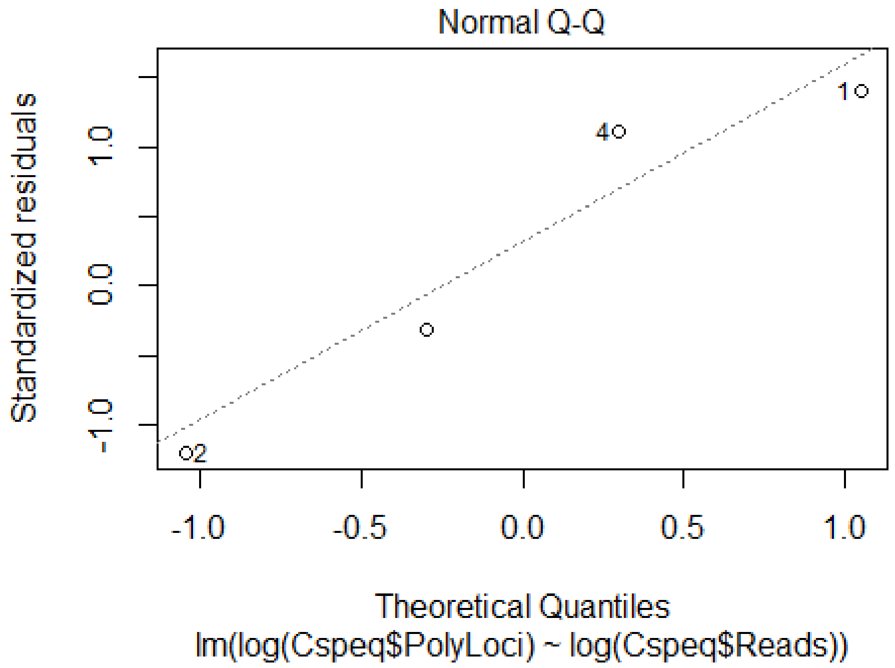

Normal Q-Q
Standardized residuals
1.0
0.0
-1.0
1
4
2
-1.0
-0.5
0.0
0.5
1.0
Theoretical Quantiles
lm(log(Cspeq$PolyLoci) ~ log(Cspeq$Reads))

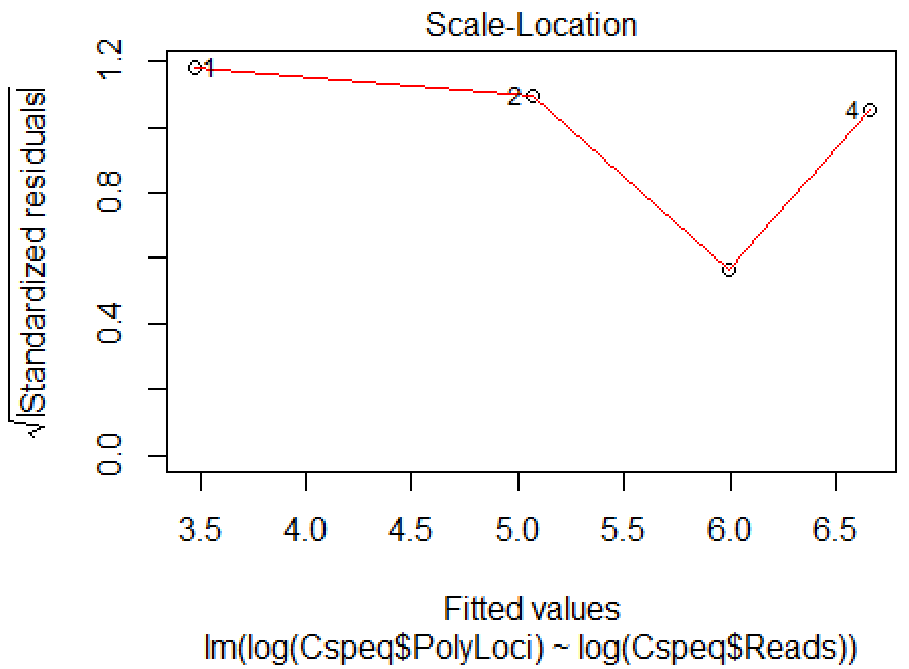

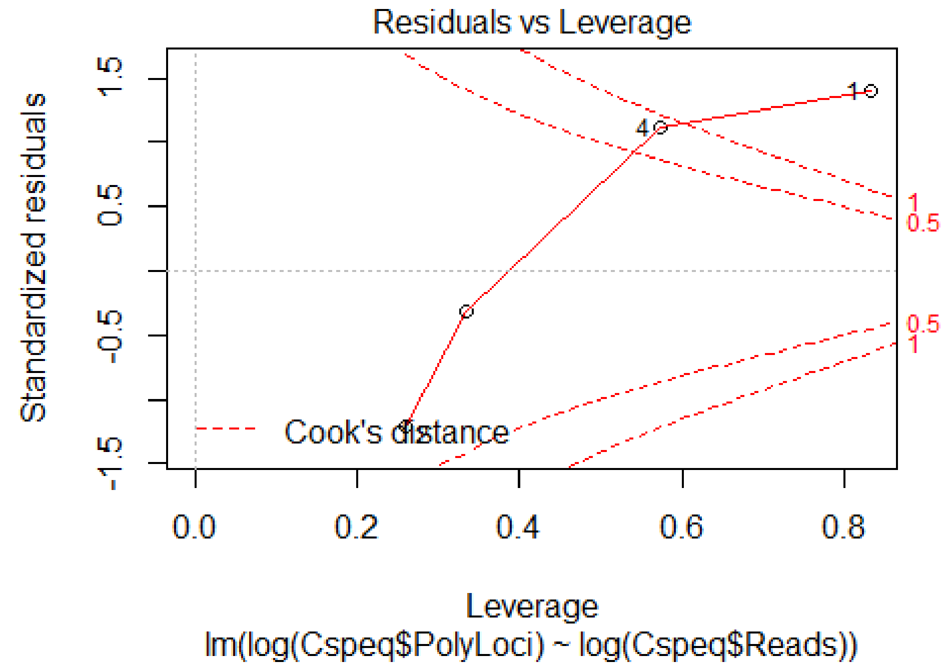

==

These are the best fitted models we chose after evaluating the Residual SE, P-values, iterations to converge, anova(residual sum Sq), Correlation y vs fitted values.

```r
mA1 <- nls(PolyLoci ~ a + b *log(Reads), start=list(a=exp(3.60543),
b=0.61202), data=Alfeq) #logarithmic model improved y = 1/(a+b*ln(x))
mB4 <- nls(PolyLoci ~ a * Reads^(b*Reads), start=list(a=exp(3.2973),
b=1.3523), data=Bcgeq)   #Geometric y = a*x^(b*x)
mC2 <- nls(PolyLoci ~ a * exp(b*Reads), start=list(a=exp(3.0635), b=2.3027),
data=Cspeq) #exponential model - improved  y=a*exp(b*x)


# Correlation to have an idea of the goodness of fit of nls models.

cor(Alfeq[2], predict(mA1))

##               [,1]
## PolyLoci 0.9924845

cor(Bcgeq[2], predict(mB4))

##               [,1]
## PolyLoci 0.9978976

cor(Cspeq[2], predict(mC2))

##               [,1]
## PolyLoci 0.9997287

# Confident intervals for the model fit.

confint(mA1)

## Waiting for profiling to be done...

##       2.5%    97.5%
## a 31.37648 45.71421
## b 17.13496 37.70768

confint(mB4)

## Waiting for profiling to be done...

##         2.5%      97.5%
## a 27.0614342 72.9796309
## b  0.1517808  0.2572599

confint(mC2)

## Waiting for profiling to be done...

##        2.5%     97.5%
## a 2.9597082 13.583893
## b 0.9435545  1.268755
```

==

Here the different nls regression models we considered do not achived a good fit.

```
mA <- nls(PolyLoci ~ a*Reads^b, start=list(a=exp(3.60543), b=0.61202),
data=Alfeq) # Power fit
mA2 <- nls(PolyLoci ~ a * exp(b*Reads), start=list(a=exp(3.60543),
b=0.61202), data=Alfeq) #exponential model - improved  y=a*exp(b*x)
mA3 <- nls(PolyLoci ~ a * b^Reads , start=list(a=exp(3.60543), b=0.61202),
data=Alfeq)     #modified power - improved y = a*b^x
mA4 <- nls(PolyLoci ~ a * Reads^(b*Reads), start=list(a=exp(3.60543),
b=0.61202), data=Alfeq)   #Geometric y = a*x^(b*x) improved more than 2 & 3
models
#mA5 <- nls(PolyLoci ~ 1/ (a + b *log(Reads)), start=list(a=exp(3.60543),
b=0.61202), data=Alfeq) # Too complex model

mB <- nls(PolyLoci ~ a*Reads^b, start=list(a=exp(3.2973), b=1.3523),
data=Bcgeq)   #Power fit
mB1 <- nls(PolyLoci ~ a + b *log(Reads), start=list(a=exp(3.2973), b=1.3523),
data=Bcgeq) #logarithmic model - not improved y = 1/(a+b*ln(x))
mB2 <- nls(PolyLoci ~ a * exp(b*Reads), start=list(a=exp(3.2973), b=1.3523),
data=Bcgeq) #exponential model - improved  y=a*exp(b*x)
mB3 <- nls(PolyLoci ~ a * b^Reads , start=list(a=exp(3.2973), b=1.3523),
data=Bcgeq)     #modified power - improved y = a*b^x
mB4 <- nls(PolyLoci ~ a * Reads^(b*Reads), start=list(a=exp(3.2973),
b=1.3523), data=Bcgeq)   #Geometric y = a*x^(b*x) improved more than 2 & 3
models



mC <- nls(PolyLoci ~ a*Reads^b, start=list(a=exp(3.0635), b=2.3027),
data=Cspeq)  #Power fit
mC1 <- nls(PolyLoci ~ a + b *log(Reads), start=list(a=exp(3.0635), b=2.3027),
data=Cspeq) #logarithmic model - not improved y = 1/(a+b*ln(x))
mC2 <- nls(PolyLoci ~ a * exp(b*Reads), start=list(a=exp(3.0635), b=2.3027),
data=Cspeq) #exponential model - improved  y=a*exp(b*x)
mC3 <- nls(PolyLoci ~ a * b^Reads , start=list(a=exp(3.0635), b=2.3027),
data=Cspeq)     #modified power - improved y = a*b^x
mC4 <- nls(PolyLoci ~ a * Reads^(b*Reads), start=list(a=exp(3.0635),
b=2.3027), data=Cspeq)   #Geometric y = a*x^(b*x) improved no more than 2 & 3
models
#mC5 <- nls(PolyLoci ~ a * Reads/(b + Reads), start=list(a=exp(3.0635),
b=2.3027), data=Cspeq)    #Saturation Growth y = ax / (b + x) did not work
```

==

LOESS models - statistical analysis

Athough LOESS models provide with an exact fit into the data, these are not very useful if your aim is to estimate model parameters which can give and indication of the sequencing depth to achieve in order to obtain a expected a determinate polymorphic loci yield.

Here are the models for each of the Enzyme data sets. It also shows an example of the code "predict(loA, loApre$a)" for predicting a determinate polymorphic loci yield expected for a determinate sequencing depth. A disadvantge is that only predicts inside the range of our experimental data.

```
#loA <- loess(Alfeq$PolyLoci~Alfeq$Reads, Alfeq)
#loApre <- data.frame(a=c(0.71, 1.43,2.15, 2.87, 3))
#loApre$loess <- predict(loA, loApre$a)

#loB <- loess(Bcgeq$PolyLoci~Bcgeq$Reads, Bcgeq)
#loBpre <- data.frame(a=c(0.71, 1.43,2.15, 2.87, 3))
#loBpre$loess <- predict(loB, loBpre$a)

#loC <- loess(Cspeq$PolyLoci~Cspeq$Reads, Cspeq)
#loCpre <- data.frame(a=c(0.71, 1.43,2.15, 2.87, 3))
#loCpre$loess <- predict(loC, loCpre$a)
```

===

These functions will be used to call the equation you want to annotate at the end of your plotting only for the nls models. You have to add the same starting values from the previous nls models above for each Enzyme.

```
# AlfI data set equation - logarithmic model y = 1/(a+b*ln(x)) did improve
model fit

nls_eqA = function(Alfeq, start=list(a=exp(3.60543), b=0.61202)){
  m = nls(PolyLoci ~ a + b * log(Reads), start=start, data=Alfeq);
  eq <- substitute(italic(y) == a + b ~ ln ~italic((x)),
list(a=format(coef(m)[1], digits=2), b=format(coef(m)[2], digits=2)))
  as.character(as.expression(eq));
}

# BcgI data set equation - Geometric y = a*x^(b*x) did improve model fit

nls_eqB = function(Bcgeq, start=list(a=exp(3.2973), b=1.3523)){
  m = nls(PolyLoci ~ a * Reads ^ (b * Reads), start=start, data=Bcgeq);
  eq <- substitute(italic(y) == a ~ italic(x)^(b ~ italic(x)),
list(a=format(coef(m)[1], digits=2), b=format(coef(m)[2], digits=2)))
  as.character(as.expression(eq));
}

# CspCI data set equation - Exponential model y=a*exp(b*x) did improve model
fit
nls_eqC = function(Cspeq, start=list(a=exp(3.0635), b=2.3027)){
  m = nls(PolyLoci ~ a * exp(b * Reads), start=start, data=Cspeq);
  eq <- substitute(italic(y) == a ~ italic(e)(b ~ italic(x)),
list(a=format(coef(m)[1], digits=2), b=format(coef(m)[2], digits=2)))
  as.character(as.expression(eq));
}
```

==

Plot your data vs fitted nls models

You might find some error when using nls function as stated in: Examples using geom_smooth(), particularly method.args...
https://github.com/tidyverse/ggplot2/issues/1510 Releases notes
https://blog.rstudio.org/2015/12/21/ggplot2-2-0-0/

Be aware that if you are using a complex equation with a simple data set, the nls might not work.

```
# Plot and add the costum code you want. I recommend to set aes and smooth
first (To see your smooth work)
# and then do all the costum changes.
#
# As you can noticed, code pre-written with a # is not run in R.

p1 <- ggplot(data=IISNP_df,
             aes(x=Reads,
                 y=PolyLoci,
                 colour = Enzyme)) +
  geom_point() +
  #facet_grid(Enzyme ~ ., scales = "free") + # use facet_grid instead
facet_wrap if you want horizontal facets.
  geom_smooth(method = "nls",
              se = FALSE,
              size = 0.8,
              method.args = list(formula = y ~ a + b * log( x),
                                 start = list(a=exp(3.60543), b=0.61202)),
              data = Alfeq, aes(x=Alfeq$Reads, y=Alfeq$PolyLoci,
colour=Alfeq$Enzyme)) +
  geom_smooth(method = "nls",
              se = FALSE,
              size = 0.8,
              method.args = list(formula = y ~ a * x ^ (b * x),
                                 start = list(a=exp(3.2973), b=1.3523)),
              data = Bcgeq, aes(x=Bcgeq$Reads, y=Bcgeq$PolyLoci,
colour=Bcgeq$Enzyme)) +
  geom_smooth(method = "nls",
              se = FALSE,
              size = 0.8,
              method.args = list(formula = y ~ a * exp(b * x),
                                 start = list(a=exp(3.0635), b=2.3027)),
              data = Cspeq, aes(x= Cspeq$Reads, y=Cspeq$PolyLoci, colour=
Cspeq$Enzyme)) +
  #geom_smooth(method = "glm", formula= y~x, se=FALSE, size=0.7) +
# For carrying out an GLMs
  #geom_smooth(method = "loess", formula= y~x, se=FALSE, size=0.7,
```

```
colour="red",                        # For carrying out LOESS
  #          data = Alfeq, aes(x=Alfeq$Reads, y=Alfeq$PolyLoci,
colour=Alfeq$Enzyme)) +
  #geom_smooth(method = "loess", formula= y~x, se=FALSE, size=0.7,
colour="red",
  #           data = Bcgeq, aes(x=Bcgeq$Reads, y=Bcgeq$PolyLoci,
colour=Bcgeq$Enzyme)) +
  #geom_smooth(method = "loess", formula= y~x, se=FALSE, size=0.7,
colour="red",
  #           data = Cspeq, aes(x=Cspeq$Reads, y=Cspeq$PolyLoci,
colour=Cspeq$Enzyme)) +
  geom_point(aes(shape = Enzyme),
             size = 3, alpha= 0.8) +
  scale_shape_manual(values=c(15,20,17)) +
  scale_color_manual(values =c("magenta3", "navyblue", "steelblue3")) +
  geom_errorbar(aes(ymin=PolyLoci-SD,
                    ymax=PolyLoci+SD),
                colour="grey30",
                size= 0.7, width= 0.08) +
  ggtitle(expression(atop(bold("A)"), ""))) +
  labs(x="Millions of reads (Mreads)") +
  labs(y="Polymorphic Loci") +
  xlim(0,6) +
  ylim(0,2400)+
  #theme(axis.title.x= element_blank()) +
  theme(text=element_text(size=17,
                          family="Arial")) +
  theme(legend.justification = c(1,0),
        legend.position = c(0.98, 0.77)) +
  theme(legend.background = element_rect(colour="black",
                                         fill="gray90")) +
  theme(legend.key = element_rect(fill = "white")) #+
  #geom_text(x=3, y=8, label = nls_eqA(Alfeq), data=Alfeq,
            #parse=TRUE, show.legend = FALSE) +
  #geom_text(x=5.5, y=450, label = nls_eqB(Bcgeq), data=Bcgeq,
            #parse=TRUE, show.legend = FALSE) +
  #geom_text(x=4.1, y=1200, label = nls_eqC(Cspeq), data=Cspeq,
            #parse=TRUE, show.legend = FALSE)

p1
```

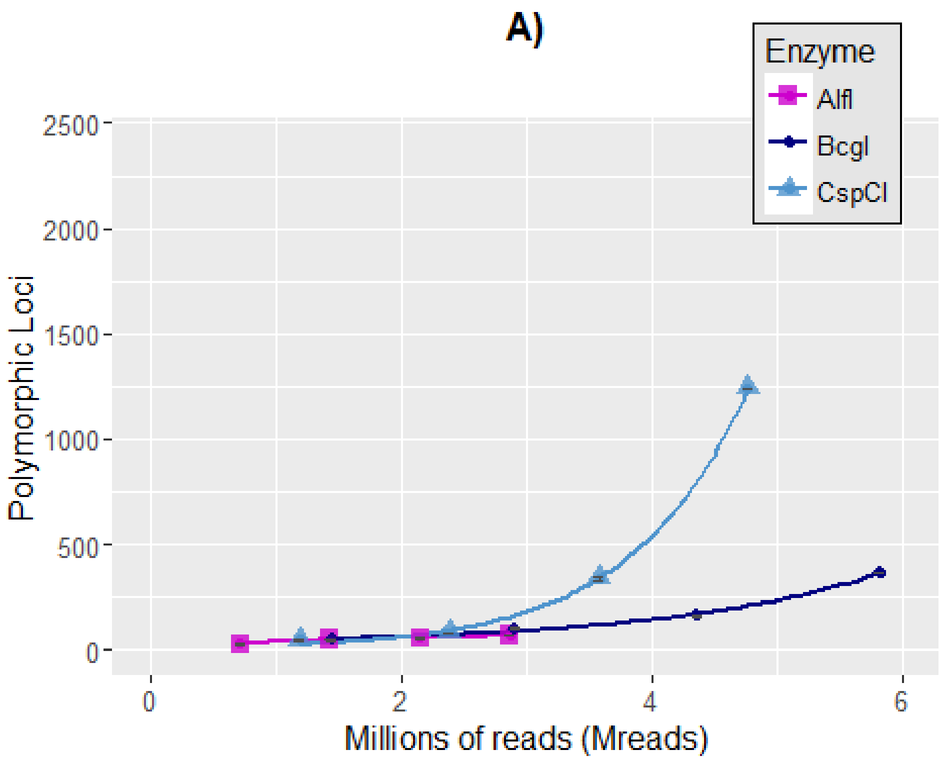

==

Read the second data set - Polymorphic loci shared by 80% of total samples

==

From here - All instructions are the same as for the 90% data set.

```
data2 <- read.csv("2SNP80.csv")
attach(data2)

## The following object is masked _by_ .GlobalEnv:
##
##     Enzyme

## The following objects are masked from data1:
##
##     Enzyme, Loci

head(data2)

##   Loci Enzyme
## 1   47 AlfI25
## 2   44 AlfI25
## 3   50 AlfI25
```

```
## 4   76 AlfI50
## 5   71 AlfI50
## 6   79 AlfI50
```

```r
# AlfI Data

MeanA25 <- mean(subset(data2, Enzyme == "AlfI25")$Loci)
SdA25 <- sd(subset(data2, Enzyme == "AlfI25")$Loci)
SeA25 <- sd(subset(data2, Enzyme == "AlfI25")$Loci)/sqrt(length(subset(data2,
Enzyme == "AlfI25")$Loci))

MeanA50 <- mean(subset(data2, Enzyme == "AlfI50")$Loci)
SdA50 <- sd(subset(data2, Enzyme == "AlfI50")$Loci)
SeA50 <- sd(subset(data2, Enzyme == "AlfI50")$Loci)/sqrt(length(subset(data2,
Enzyme == "AlfI50")$Loci))


MeanA75 <- mean(subset(data2, Enzyme == "AlfI75")$Loci)
SdA75 <- sd(subset(data2, Enzyme == "AlfI75")$Loci)
SeA75 <- sd(subset(data2, Enzyme == "AlfI75")$Loci)/sqrt(length(subset(data2,
Enzyme == "AlfI75")$Loci))

MeanA100 <- mean(subset(data2, Enzyme == "AlfI100")$Loci)
SdA100 <- sd(subset(data2, Enzyme == "AlfI100")$Loci)
SeA100 <- sd(subset(data2, Enzyme ==
"AlfI100")$Loci)/sqrt(length(subset(data2, Enzyme == "AlfI100")$Loci))

# BcgI data

MeanB25 <- mean(subset(data2, Enzyme == "BcgI25")$Loci)
SdB25 <- sd(subset(data2, Enzyme == "BcgI25")$Loci)
SeB25 <- sd(subset(data2, Enzyme == "BcgI25")$Loci)/sqrt(length(subset(data2,
Enzyme == "BcgI25")$Loci))

MeanB50 <- mean(subset(data2, Enzyme == "BcgI50")$Loci)
SdB50 <- sd(subset(data2, Enzyme == "BcgI50")$Loci)
SeB50 <- sd(subset(data2, Enzyme == "BcgI50")$Loci)/sqrt(length(subset(data2,
Enzyme == "BcgI50")$Loci))

MeanB75 <- mean(subset(data2, Enzyme == "BcgI75")$Loci)
SdB75 <- sd(subset(data2, Enzyme == "BcgI75")$Loci)
SeB75 <- sd(subset(data2, Enzyme == "BcgI75")$Loci)/sqrt(length(subset(data2,
Enzyme == "BcgI75")$Loci))

MeanB100 <- mean(subset(data2, Enzyme == "BcgI100")$Loci)
SdB100 <- sd(subset(data2, Enzyme == "BcgI100")$Loci)
SeB100 <- sd(subset(data2, Enzyme ==
"BcgI100")$Loci)/sqrt(length(subset(data2, Enzyme == "BcgI100")$Loci))
```

```
# CspCI data

MeanC25 <- mean(subset(data2, Enzyme == "CspCI25")$Loci)
SdC25 <- sd(subset(data2, Enzyme == "CspCI25")$Loci)
SeC25 <- sd(subset(data2, Enzyme ==
"CspCI25")$Loci)/sqrt(length(subset(data2, Enzyme == "CspCI25")$Loci))

MeanC50 <- mean(subset(data2, Enzyme == "CspCI50")$Loci)
SdC50 <- sd(subset(data2, Enzyme == "CspCI50")$Loci)
SeC50 <- sd(subset(data2, Enzyme ==
"CspCI50")$Loci)/sqrt(length(subset(data2, Enzyme == "CspCI50")$Loci))

MeanC75 <- mean(subset(data2, Enzyme == "CspCI75")$Loci)
SdC75 <- sd(subset(data2, Enzyme == "CspCI75")$Loci)
SeC75 <- sd(subset(data2, Enzyme ==
"CspCI75")$Loci)/sqrt(length(subset(data2, Enzyme == "CspCI75")$Loci))

MeanC100 <- mean(subset(data2, Enzyme == "CspCI100")$Loci)
SdC100 <- sd(subset(data2, Enzyme == "CspCI100")$Loci)
SeC100 <- sd(subset(data2, Enzyme ==
"CspCI100")$Loci)/sqrt(length(subset(data2, Enzyme == "CspCI100")$Loci))

# Data frame

Enzyme <- c("AlfI", "AlfI", "AlfI", "AlfI", "BcgI", "BcgI", "BcgI","BcgI",
"CspCI", "CspCI", "CspCI", "CspCI")
PolyLoci2 <- c(MeanA25, MeanA50, MeanA75, MeanA100, MeanB25, MeanB50,
MeanB75, MeanB100, MeanC25, MeanC50, MeanC75, MeanC100)
Reads2 <- c(718061, 1436126, 2154183, 2872241, 1455376, 2910750, 4366118,
5821489, 1192825, 2385650, 3578472, 4771292)/1000000
SD2 <- c(SdA25, SdA50, SdA75, SdA100, SdB25, SdB50, SdB75, SdB100, SdC25,
SdC50, SdC75, SdC100)
SE2 <- c(SeA25, SeA50, SeA75, SeA100, SeB25, SeB50, SeB75, SeB100, SeC25,
SeC50, SeC75, SeC100)
IISNP_df2 <- data.frame(Reads2, PolyLoci2, SD2, SE2, Enzyme)

IISNP_df2

##      Reads2  PolyLoci2       SD2       SE2 Enzyme
## 1  0.718061   47.00000  3.000000  1.732051   AlfI
## 2  1.436126   75.33333  4.041452  2.333333   AlfI
## 3  2.154183   99.00000  1.732051  1.000000   AlfI
## 4  2.872241  186.00000  0.000000  0.000000   AlfI
## 5  1.455376   78.33333  3.055050  1.763834   BcgI
## 6  2.910750  149.00000  6.082763  3.511885   BcgI
## 7  4.366118  331.00000 10.440307  6.027714   BcgI
## 8  5.821489  899.00000  0.000000  0.000000   BcgI
## 9  1.192825   65.00000  6.928203  4.000000  CspCI
## 10 2.385650  154.33333  5.859465  3.382964  CspCI
```

```
## 11 3.578472  995.00000 23.430749 13.527749  CspCI
## 12 4.771292 2289.00000  0.000000  0.000000  CspCI
```

===

Subset in new data frames the data for each Enzyme, this information will be use to annote each Enzyme Polymorphic loci vs Reads equations.

```
Alfeq2 <- subset(IISNP_df2, Enzyme=="AlfI")
Bcgeq2 <- subset(IISNP_df2, Enzyme == "BcgI")
Cspeq2 <- subset(IISNP_df2, Enzyme == "CspCI")
```

===

nls models - statistical analysis

To calculate the parameters a and b for your nls models:

```
parmA2 <- lm(log(Alfeq2$PolyLoci2)~log(Alfeq2$Reads2))
anova(update(parmA2, ~ . -1), parmA2)

## Analysis of Variance Table
##
## Model 1: log(Alfeq2$PolyLoci2) ~ log(Alfeq2$Reads2) - 1
## Model 2: log(Alfeq2$PolyLoci2) ~ log(Alfeq2$Reads2)
##   Res.Df    RSS Df Sum of Sq      F   Pr(>F)
## 1      3 37.130
## 2      2  0.081  1    37.049 918.09 0.001087 **
## ---
## Signif. codes:  0 '***' 0.001 '**' 0.01 '*' 0.05 '.' 0.1 ' ' 1

plot(parmA2)
```

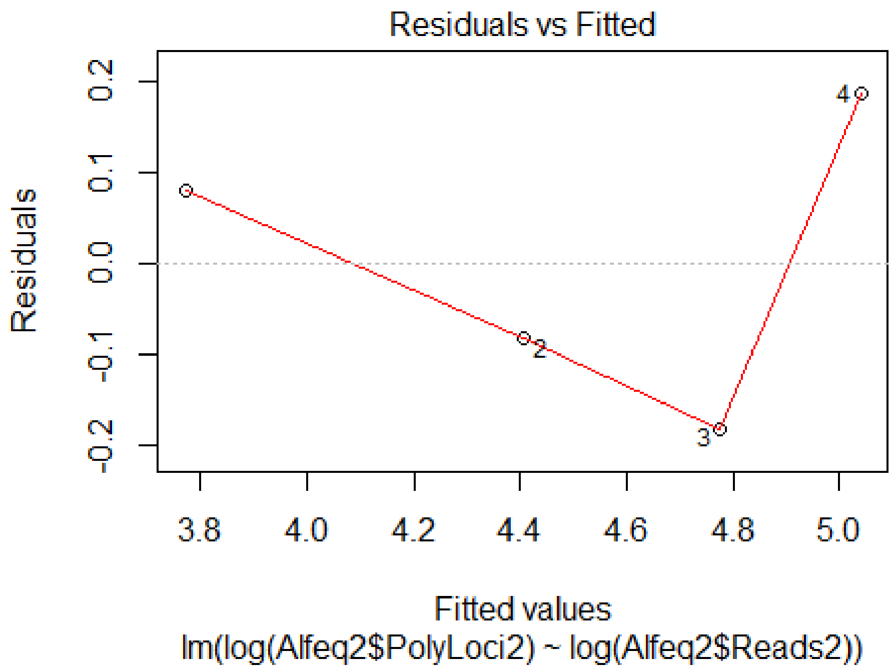
Residuals vs Fitted
Residuals
0.2
0.1
0.0
-0.1
-0.2
3.8
4.0
4.2
4.4
4.6
4.8
5.0
4
2
3
Fitted values
lm(log(Alfeq2$PolyLoci2) ~ log(Alfeq2$Reads2))

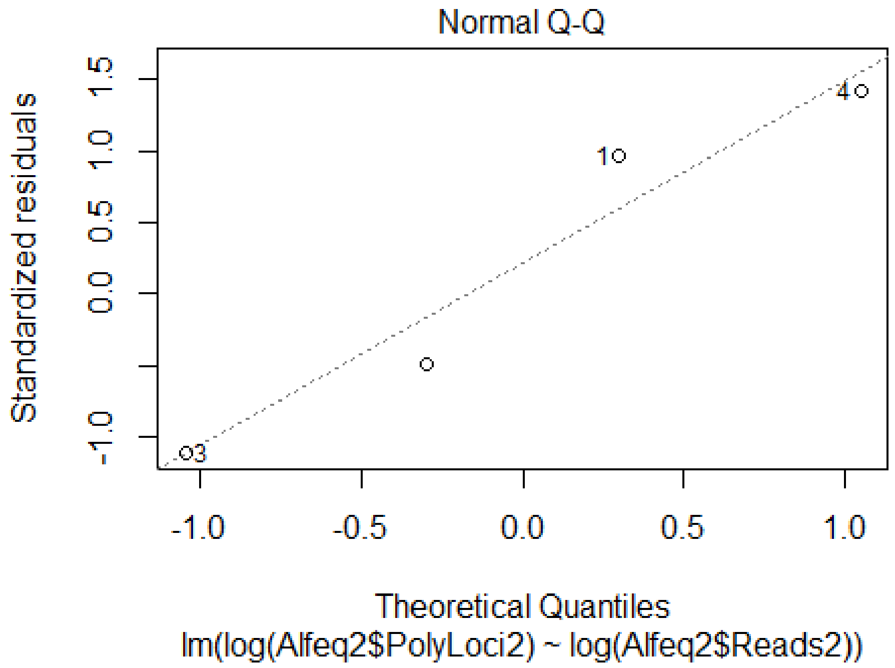
Normal Q-Q
Standardized residuals
1.5
1.0
0.5
0.0
-1.0
-1.0
-0.5
0.0
0.5
1.0
4
1
3
Theoretical Quantiles
lm(log(Alfeq2$PolyLoci2) ~ log(Alfeq2$Reads2))

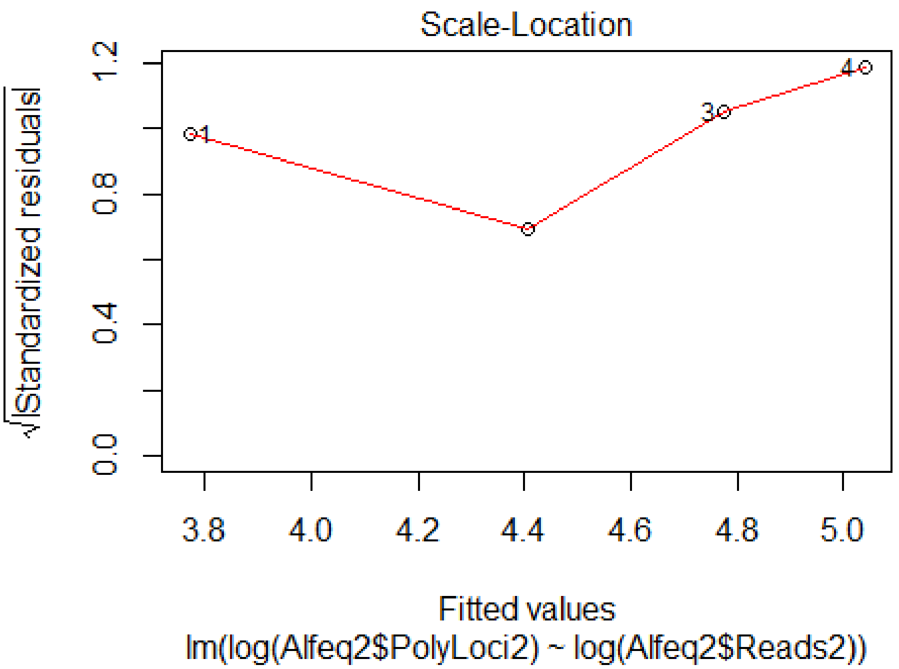

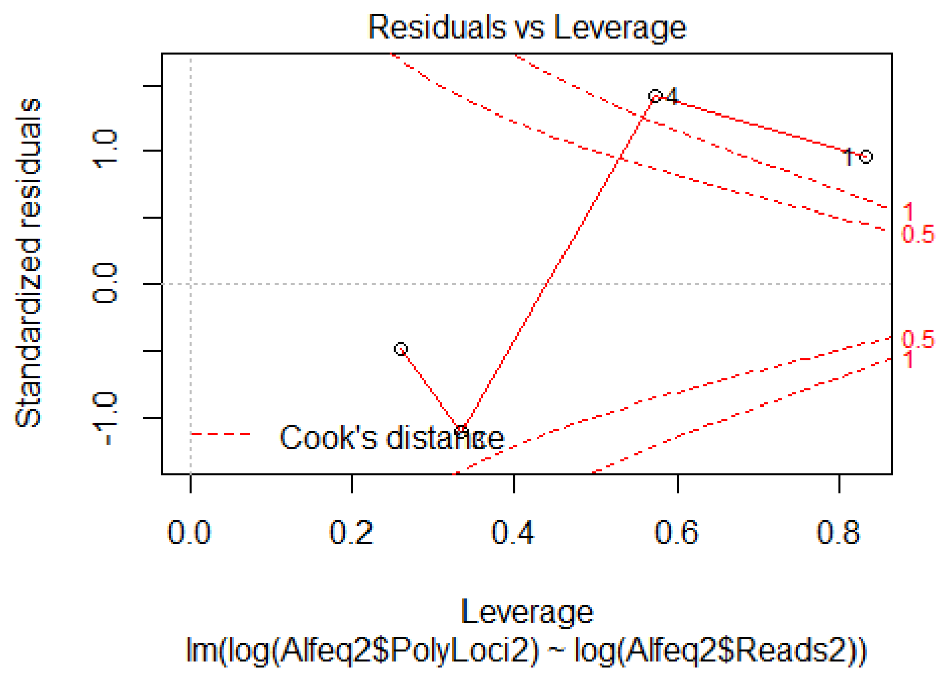


```
parmB2 <- lm(log(Bcgeq2$PolyLoci2)~log(Bcgeq2$Reads2))
anova(update(parmB2, ~ . -1), parmB2)
```

```
## Analysis of Variance Table
##
## Model 1: log(Bcgeq2$PolyLoci2) ~ log(Bcgeq2$Reads2) - 1
## Model 2: log(Bcgeq2$PolyLoci2) ~ log(Bcgeq2$Reads2)
##   Res.Df    RSS Df Sum of Sq      F  Pr(>F)
## 1      3 8.5296
## 2      2 0.2821  1    8.2476 58.481 0.01667 *
## ---
## Signif. codes:  0 '***' 0.001 '**' 0.01 '*' 0.05 '.' 0.1 ' ' 1
```

```
plot(parmB2)
```

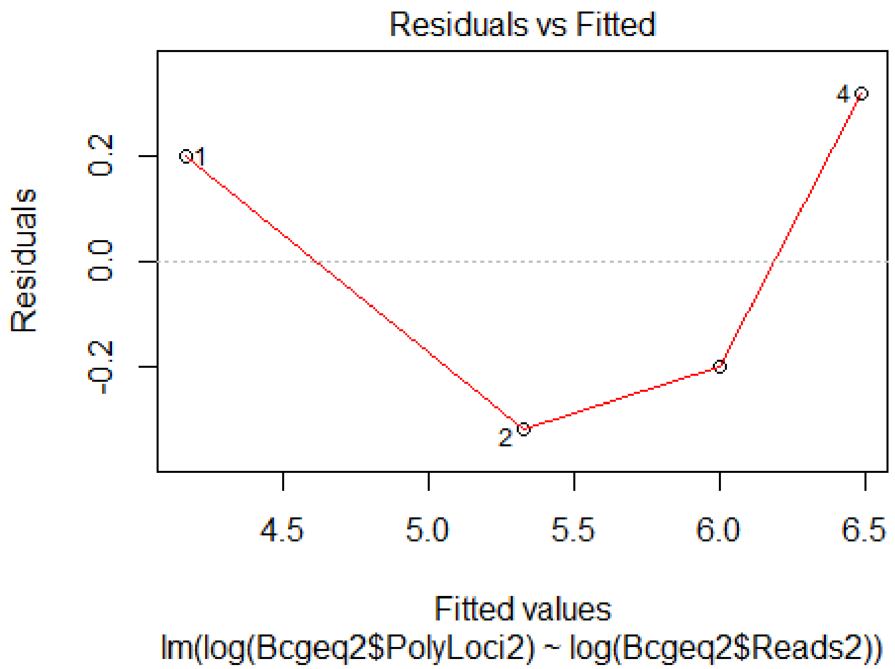

Residuals vs Fitted
Residuals
0.2
0.0
-0.2
1
2
4
4.5
5.0
5.5
6.0
6.5
Fitted values
lm(log(Bcgeq2$PolyLoci2) ~ log(Bcgeq2$Reads2))

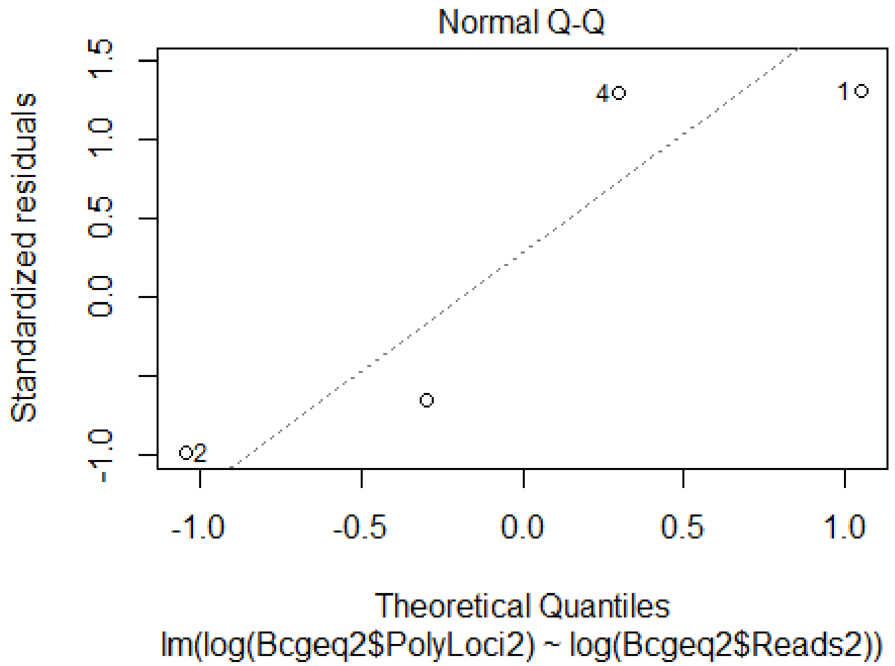

Normal Q-Q
Standardized residuals
1.5
1.0
0.5
0.0
-1.0
4
1
2
-1.0
-0.5
0.0
0.5
1.0
Theoretical Quantiles
lm(log(Bcgeq2$PolyLoci2) ~ log(Bcgeq2$Reads2))

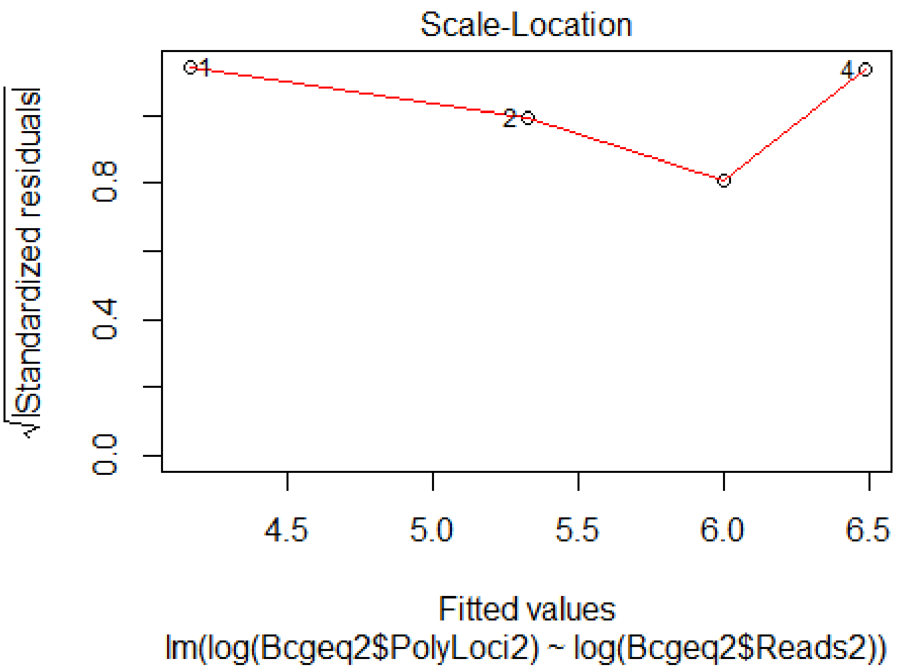

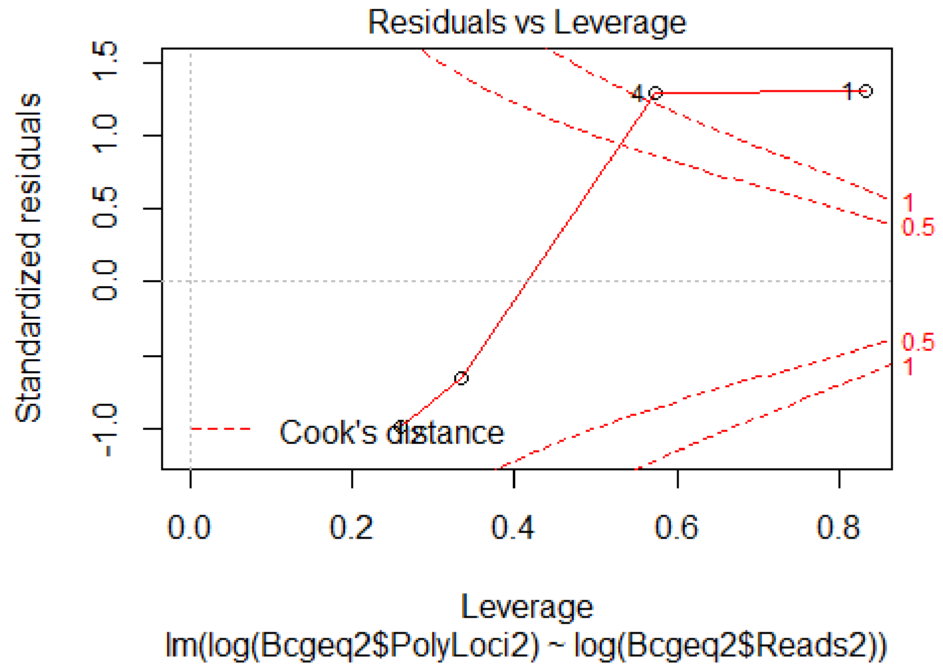


```
parmC2 <- lm(log(Cspeq2$PolyLoci2)~log(Cspeq2$Reads2))
anova(update(parmC2, ~ . -1), parmC2)
```

```
## Analysis of Variance Table
##
## Model 1: log(Cspeq2$PolyLoci2) ~ log(Cspeq2$Reads2) - 1
## Model 2: log(Cspeq2$PolyLoci2) ~ log(Cspeq2$Reads2)
##   Res.Df     RSS Df Sum of Sq      F  Pr(>F)
## 1      3 10.9858
## 2      2  0.5891  1    10.397 35.299 0.02718 *
## ---
## Signif. codes:  0 '***' 0.001 '**' 0.01 '*' 0.05 '.' 0.1 ' ' 1

plot(parmC2)
```

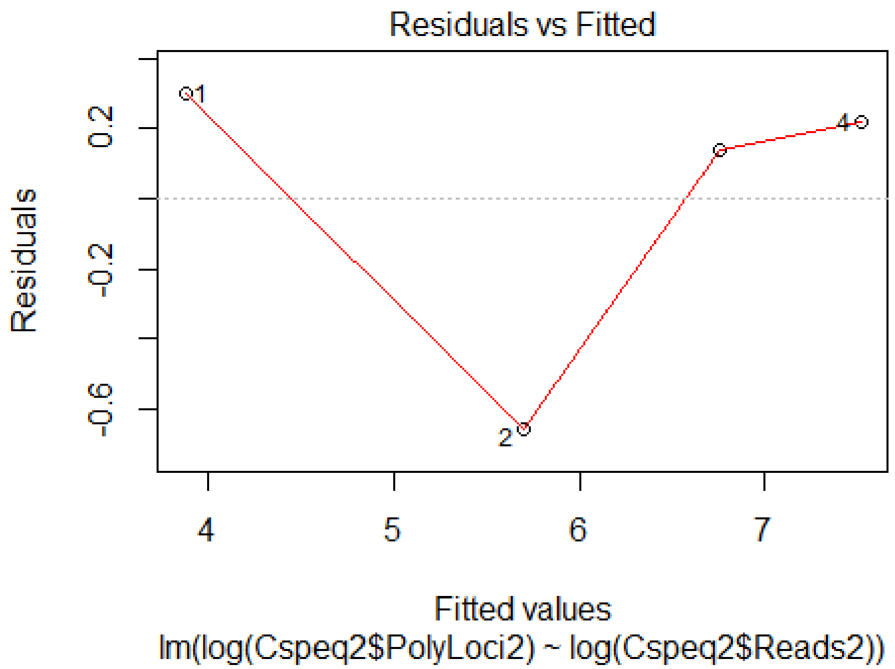
Residuals vs Fitted
Residuals
0.2
-0.2
-0.6
1
2
4
4
5
6
7
Fitted values
lm(log(Cspeq2$PolyLoci2) ~ log(Cspeq2$Reads2))

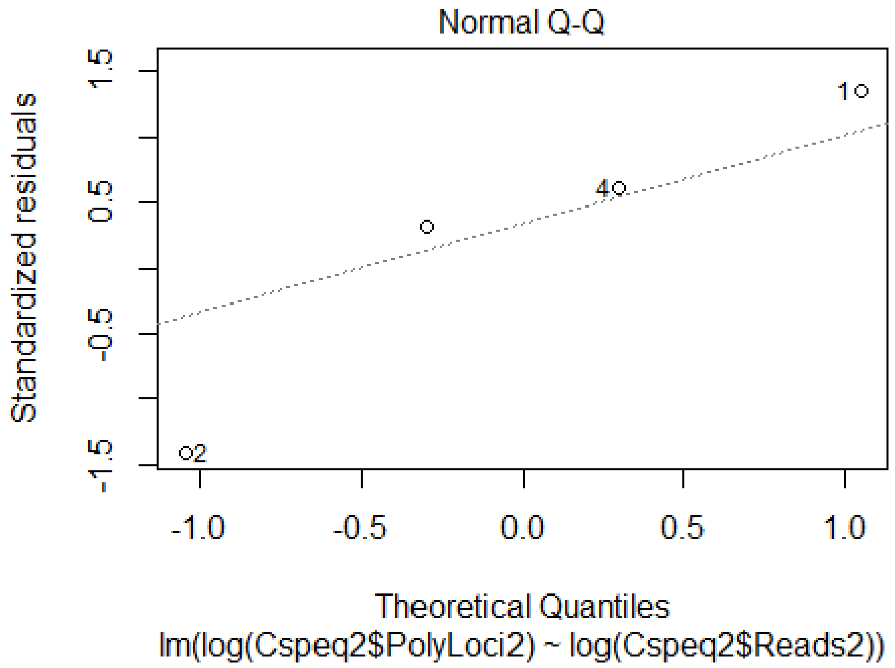
Normal Q-Q
Standardized residuals
1.5
0.5
-0.5
-1.5
1
4
2
-1.0
-0.5
0.0
0.5
1.0
Theoretical Quantiles
lm(log(Cspeq2$PolyLoci2) ~ log(Cspeq2$Reads2))

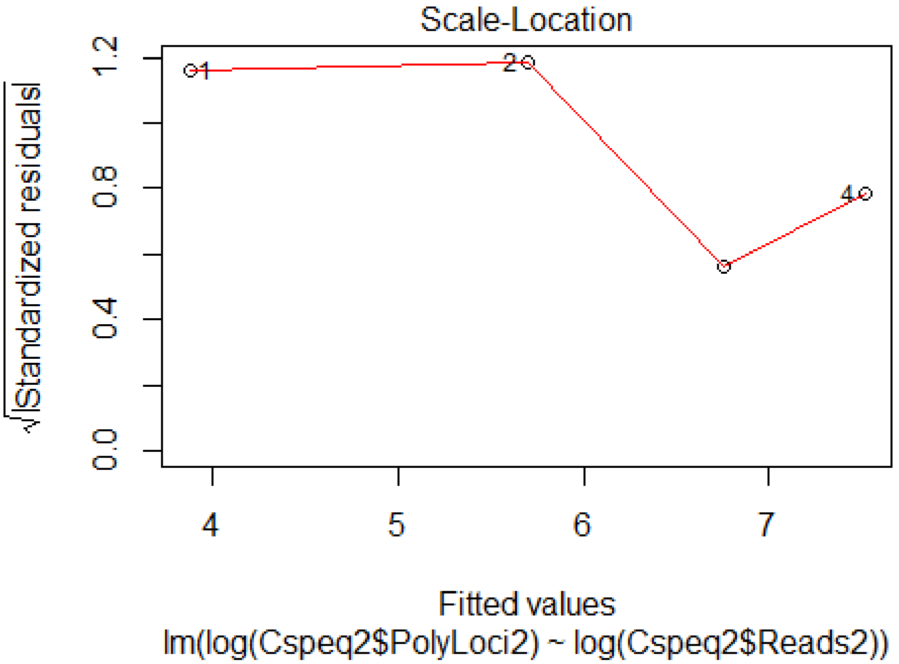

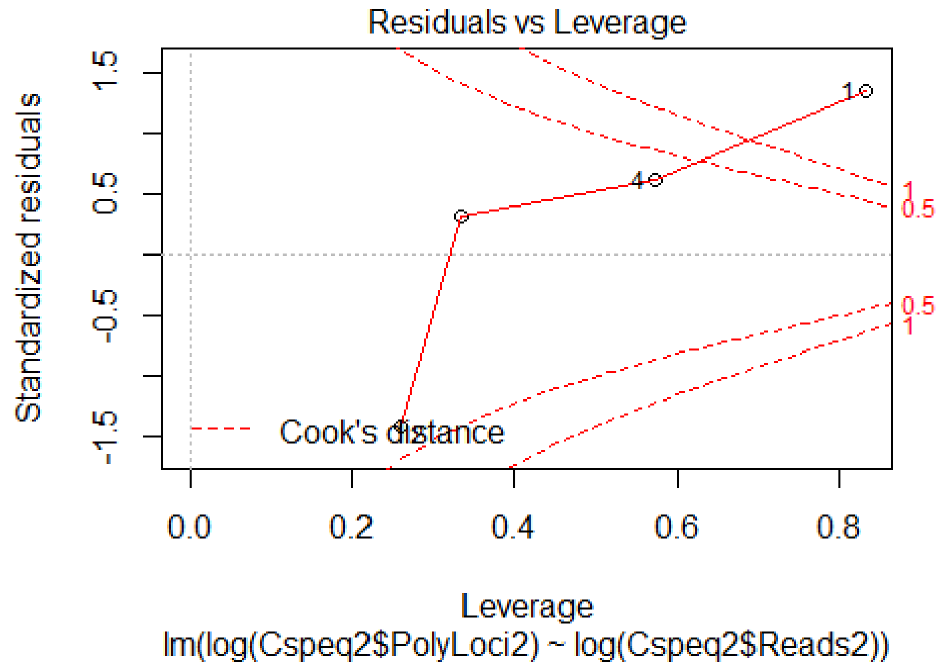

Best fitted models after evaluating Residual SE, P-values, iterations to converge, anova(residual sum Sq), Correlation y vs fitted values .

```
mmA4 <- nls(PolyLoci2 ~ a * Reads2^(b*Reads2), start=list(a=exp(4.07),
b=0.9157), data=Alfeq2)   #Geometric y = a*x^(b*x) improved more than 2 & 3
models
mmB4 <- nls(PolyLoci2 ~ a * Reads2^(b*Reads2), start=list(a=exp(3.5315),
b=1.6761), data=Bcgeq2)   #Geometric y = a*x^(b*x) improved more than 2 & 3
models
mmC <- nls(PolyLoci2 ~ a*Reads2^b, start=list(a=exp(3.4113), b=2.6283),
data=Cspeq2)  #Power fit



#some correlations of nls models

cor(Alfeq2[2], predict(mmA4))

##                [,1]
## PolyLoci2 0.9942596

cor(Bcgeq2[2], predict(mmB4))

##                [,1]
## PolyLoci2 0.9999118

cor(Cspeq2[2], predict(mmC))

##                [,1]
## PolyLoci2 0.9974096

confint(mmA4)

## Waiting for profiling to be done...

##         2.5%      97.5%
## a 34.5575103 75.8972686
## b  0.2646562  0.5704376

confint(mmB4)

## Waiting for profiling to be done...

##         2.5%      97.5%
## a 54.7729736 75.6041980
## b  0.2404015  0.2735109

#confint(mmC2)
```

===

```
# Different nls regression models whihc fail to provide a good fit.

mmA1 <- nls(PolyLoci2 ~ a + b *log(Reads2), start=list(a=exp(4.07),
b=0.9157), data=Alfeq2) #logarithmic model improved y = 1/(a+b*ln(x))
```

```
mmA0 <- nls(PolyLoci2 ~ a* Reads2^b, start=list(a=exp(4.07), b=0.9157),
data=Alfeq2)
mmA <- nls(PolyLoci2 ~ a*Reads2^b, start=list(a=exp(4.07), b=0.9157),
data=Alfeq2) # Power fit
mmA2 <- nls(PolyLoci2 ~ a * exp(b*Reads2), start=list(a=exp(4.07), b=0.9157),
data=Alfeq2) #exponential model - improved  y=a*exp(b*x)
mmA3 <- nls(PolyLoci2 ~ a * b^Reads2 , start=list(a=exp(4.07), b=0.9157),
data=Alfeq2)     #modified power - improved y = a*b^x
mmA4 <- nls(PolyLoci2 ~ a * Reads2^(b*Reads2), start=list(a=exp(4.07),
b=0.9157), data=Alfeq2)   #Geometric y = a*x^(b*x) improved more than 2 & 3
models


mmB <- nls(PolyLoci2 ~ a*Reads2^b, start=list(a=exp(3.5315), b=1.6761),
data=Bcgeq2)   #Power fit
mmB1 <- nls(PolyLoci2 ~ a + b *log(Reads2), start=list(a=exp(3.5315),
b=1.6761), data=Bcgeq2) #logarithmic model - not improved y = 1/(a+b*ln(x))
mmB2 <- nls(PolyLoci2 ~ a * exp(b*Reads2), start=list(a=exp(3.5315),
b=1.6761), data=Bcgeq2) #exponential model - improved  y=a*exp(b*x)
mmB3 <- nls(PolyLoci2 ~ a * b^Reads2 , start=list(a=exp(3.5315), b=1.6761),
data=Bcgeq2)     #modified power - improved y = a*b^x
mmB4 <- nls(PolyLoci2 ~ a * Reads2^(b*Reads2), start=list(a=exp(3.5315),
b=1.6761), data=Bcgeq2)   #Geometric y = a*x^(b*x) improved more than 2 & 3
models



mmC <- nls(PolyLoci2 ~ a*Reads2^b, start=list(a=exp(3.4113), b=2.6283),
data=Cspeq2)  #Power fit
mmC1 <- nls(PolyLoci2 ~ a + b *log(Reads2), start=list(a=exp(3.4113),
b=2.6283), data=Cspeq2) #logarithmic model - not improved y = 1/(a+b*ln(x))
mmC2 <- nls(PolyLoci2 ~ a * exp(b*Reads2), start=list(a=exp(3.4113),
b=2.6283), data=Cspeq2) #exponential model - improved  y=a*exp(b*x)
mmC3 <- nls(PolyLoci2 ~ a * b^Reads2 , start=list(a=exp(3.4113), b=2.6283),
data=Cspeq2)     #modified power - improved y = a*b^x
mmC4 <- nls(PolyLoci2 ~ a * Reads2^(b*Reads2), start=list(a=exp(3.4113),
b=2.6283), data=Cspeq2)   #Geometric y = a*x^(b*x) improved no more than 2 &
3 models
mmC5 <- nls(PolyLoci2 ~ (a + b/Reads2 ), start=list(a=exp(3.4113), b=2.6283),
data=Cspeq2)# Hyperbolic Fit y = a + b/x
```

==========================

Try LOESS models - statistical analysis

only predict Loci inside the range of data given. Out of these range it can predict as it is a local modelling.

```
#loA2 <- loess(Alfeq2$PolyLoci~Alfeq2$Reads, Alfeq2)
#loApre2 <- data.frame(a=c(0.71, 1.43,2.15, 2.87, 3))
#loApre2$loess2 <- predict(loA2, loApre2$a)

#loB2 <- loess(Bcgeq2$PolyLoci~Bcgeq2$Reads, Bcgeq2)
#loBpre2 <- data.frame(a=c(0.71, 1.43,2.15, 2.87, 3))
#loBpre2$loess2 <- predict(loB2, loBpre2$a)

#loC2 <- loess(Cspeq2$PolyLoci~Cspeq2$Reads, Cspeq2)
#loCpre2 <- data.frame(a=c(1.192825, 2.385650,3.578472, 4, 4.771292))
#loCpre2$loess2 <- predict(loC2, loCpre2$a)
```

===

These functions will be used to call the equation you want to annotate at the end of your plotting only for the nls models. You have to add the same starting values from the previous nls models above for each Enzyme.

```
nls_eqA2 = function(Alfeq2, start=list(a=exp(4.07), b=0.9157)){
  m = nls(PolyLoci2 ~ a * Reads2^(b*Reads2), start=start, data=Alfeq2);
  eq <- substitute(italic(y) == a ~ italic(x)^(b~italic(x)),
list(a=format(coef(m)[1], digits=2), b=format(coef(m)[2], digits=2)))
  as.character(as.expression(eq));
}

nls_eqB2 = function(Bcgeq2, start=list(a=exp(3.5315), b=1.6761)){
  m = nls(PolyLoci2 ~ a * Reads2^(b*Reads2), start=start, data=Bcgeq2);
  eq <- substitute(italic(y) == a ~ italic(x)^(b~italic(x)),
list(a=format(coef(m)[1], digits=2), b=format(coef(m)[2], digits=2)))
  as.character(as.expression(eq));
}

nls_eqC2 = function(Cspeq2, start=list(a=exp(3.4113), b=2.6283)){
  m = nls(PolyLoci2 ~ a*Reads2^b, start=start, data=Cspeq2);
  eq <- substitute(italic(y) == a ~ italic(x)^b, list(a=format(coef(m)[1],
digits=2), b=format(coef(m)[2], digits=2)))
  as.character(as.expression(eq));
}
```

===

Plot your data vs fitted nls models

```
# Plot and add the costum code you want. I recommend to set aes and smooth
first (To see your smooth work)
# and then do all the costum changes.
#

p2 <- ggplot(data=IISNP_df2,
             aes(x=Reads2,
```

```
                y=PolyLoci2,
                colour = Enzyme)) +
  geom_point() +
  #facet_grid(Enzyme ~ ., scales = "free") + # use facet_grid instead
facet_wrap if you want horizontal facets.
  geom_smooth(method = "nls",
              se = FALSE,
              size = 0.8,
              method.args = list(formula = y ~ a * x^(b * x),
                                 start = list(a=exp(4.07), b=0.9157)),
              data = Alfeq2, aes(x=Alfeq2$Reads2, y=Alfeq2$PolyLoci2,
colour=Alfeq2$Enzyme)) +
  geom_smooth(method = "nls",
              se = FALSE,
              size = 0.8,
              method.args = list(formula = y ~ a * x^(b * x),
                                 start = list(a=exp(3.5315), b=1.6761)),
              data = Bcgeq2, aes(x=Bcgeq2$Reads2, y=Bcgeq2$PolyLoci2,
colour=Bcgeq2$Enzyme)) +
  geom_smooth(method = "nls",
              se = FALSE,
              size = 0.8,
              method.args = list(formula = y ~ a * x^b,
                                 start = list(a=exp(3.0635), b=2.3027)),
              data = Cspeq2, aes(x=Cspeq2$Reads2, y=Cspeq2$PolyLoci2,
colour=Cspeq2$Enzyme)) +
  #geom_smooth(method = "glm", formula= y~x, se=FALSE, size=0.7) +
  #geom_smooth(method = "loess", formula= y~x, se=FALSE, size=0.7,
colour="red",
  #         data = Alfeq2, aes(x=Alfeq2$Reads, y=Alfeq2$PolyLoci,
colour=Alfeq2$Enzyme)) +
  #geom_smooth(method = "loess", formula= y~x, se=FALSE, size=0.7,
colour="red",
  #           data = Bcgeq2, aes(x=Bcgeq2$Reads, y=Bcgeq2$PolyLoci,
colour=Bcgeq2$Enzyme)) +
  #geom_smooth(method = "loess", formula= y~x, se=FALSE, size=0.7,
colour="red",
  #           data = Cspeq2, aes(x=Cspeq2$Reads, y=Cspeq2$PolyLoci,
colour=Cspeq2$Enzyme)) +
  geom_point(aes(shape = Enzyme),
             size = 3, alpha= 0.8) +
  scale_shape_manual(values=c(15,20,17)) +
  scale_color_manual(values =c("magenta3", "navyblue", "steelblue3")) +
  geom_errorbar(aes(ymin=PolyLoci2-SD,
                    ymax=PolyLoci2+SD),
                colour="grey30",
                size= 0.7, width= 0.08) +
  ggtitle(expression(atop(bold("B)"), ""))) +
  labs(x="Millions of reads (Mreads)") +
  labs(y="Polymorphic Loci") +
```

```
  xlim(0,6) +
  ylim(0,2400)+
  theme(axis.title.y = element_blank()) +
  theme(text=element_text(size=17,
                          family="Arial")) +
  theme(legend.justification = c(1,0),
        legend.position = c(0.98, 0.77)) +
  theme(legend.background = element_rect(colour="black",
                                         fill="gray90")) +
  theme(legend.key = element_rect(fill = "white")) #+
  #geom_text(x=3.2, y=47, label = nls_eqA2(Alfeq2), data=Alfeq2,
            #parse=TRUE, show.legend = FALSE) +
  #geom_text(x=5.1, y=900, label = nls_eqB2(Bcgeq2), data=Bcgeq2,
            #parse=TRUE, show.legend = FALSE) +
  #geom_text(x=3.9, y=2100, label = nls_eqC2(Cspeq2), data=Cspeq2,
            #parse=TRUE, show.legend = FALSE)

p2
```

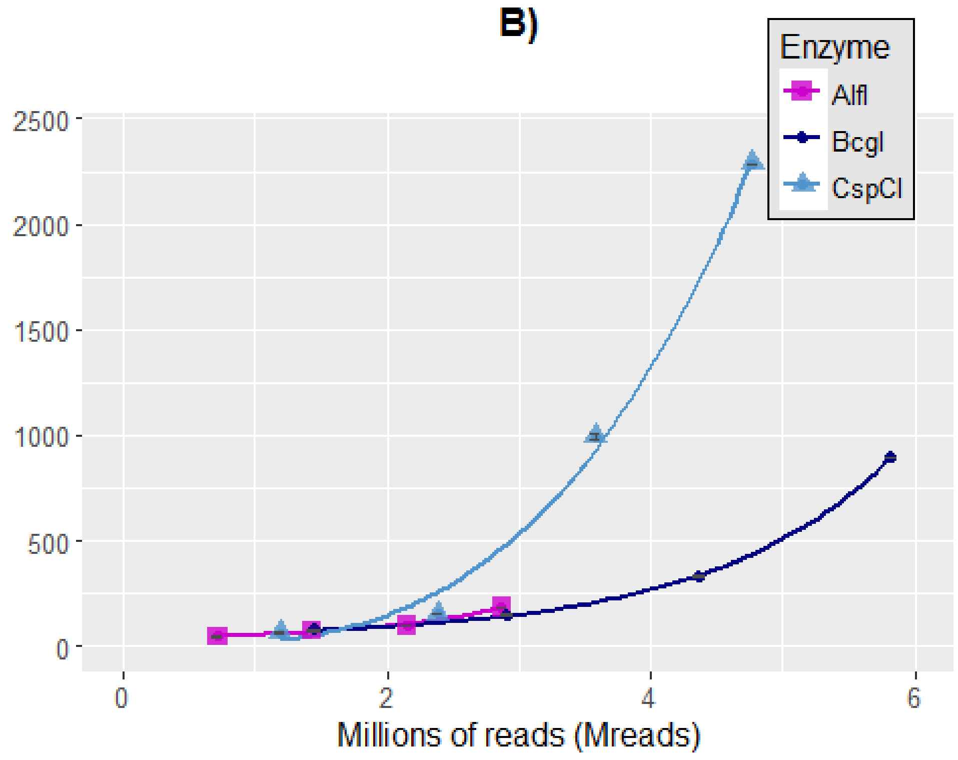

==

Multiplotting plot 1 and 2

```
multiplot <- function(..., plotlist=NULL, file, cols=1, layout=NULL) {
  library(grid)

  # Make a list from the ... arguments and plotlist
```

```
  plots <- c(list(...), plotlist)

  numPlots = length(plots)

  # If layout is NULL, then use 'cols' to determine layout
  if (is.null(layout)) {
    # Make the panel
    # ncol: Number of columns of plots
    # nrow: Number of rows needed, calculated from # of cols
    layout <- matrix(seq(1, cols * ceiling(numPlots/cols)),
                     ncol = cols, nrow = ceiling(numPlots/cols))
  }

  if (numPlots==1) {
    print(plots[[1]])

  } else {
    # Set up the page
    grid.newpage()
    pushViewport(viewport(layout = grid.layout(nrow(layout), ncol(layout))))

    # Make each plot, in the correct location
    for (i in 1:numPlots) {
      # Get the i,j matrix positions of the regions that contain this subplot
      matchidx <- as.data.frame(which(layout == i, arr.ind = TRUE))

      print(plots[[i]], vp = viewport(layout.pos.row = matchidx$row,
                                      layout.pos.col = matchidx$col))
    }
  }
}

multiplot(p1, p2, cols=2)
```

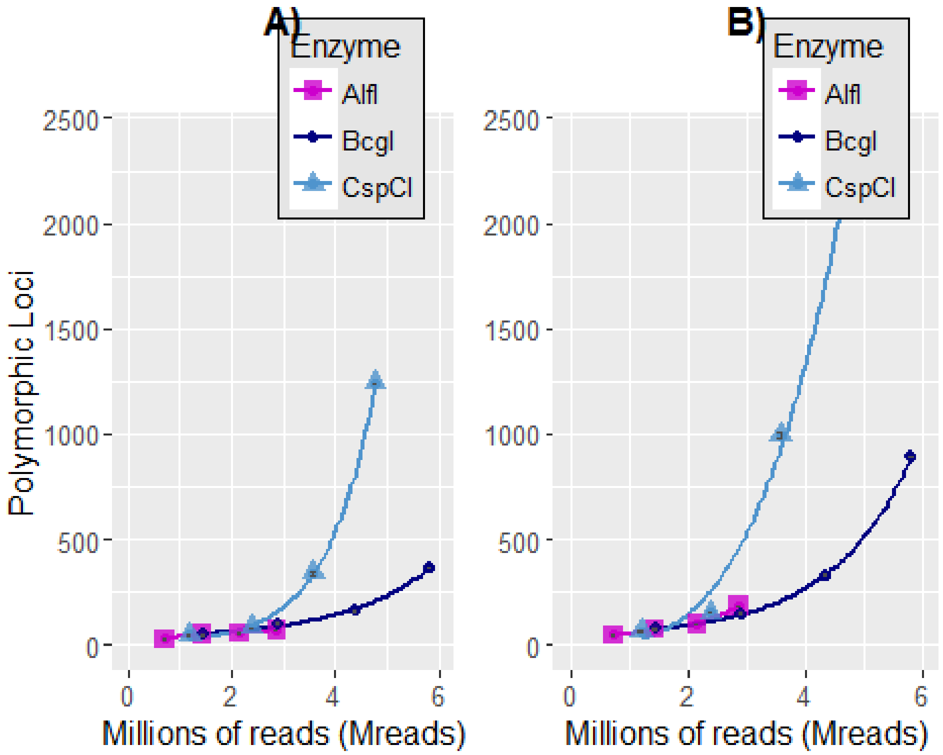
A)
B)
Enzyme
AlfI
BcgI
CspCI
Polymorphic Loci
0
500
1000
1500
2000
2500
0
2
4
6
Millions of reads (Mreads)
